# Role and mechanism of nitric oxide-regulated cGAS/STING pathway-mediated inflammatory response in hypoglycemia-induced coronary artery endothelial cell injury

**DOI:** 10.1371/journal.pone.0350983

**Published:** 2026-06-26

**Authors:** Wenping Luo, Xiao Wei, Qian Xiao

**Affiliations:** Department of Cardiology, Bishan hospital of Chongqing medical university, Bishan Hospital of Chongqing, China; UMass Chan Medical School Department of Medicine: University of Massachusetts Chan Medical School Department of Medicine, UNITED STATES OF AMERICA

## Abstract

**Background:**

Hypoglycemia in diabetes markedly increases the risk of coronary artery events, but the mechanism by which it damages vascular endothelium through nitric oxide (NO) regulation of innate immune pathways remains unclear.

**Objective:**

This study aimed to investigate the mechanism by which NO may induce vascular endothelial injury under hypoglycemic conditions, potentially through activation of the cyclic GMP-AMP synthase/stimulator of interferon genes (cGAS/STING) pathway.

**Methods:**

The mouse model of diabetic hypoglycemia and a primary endothelial cell model of low-high glucose cycling were constructed. Gene knockout, molecular biology, and functional assay were adopted.

**Results:**

In the hypoglycemia group, endothelial cell NO levels increased by 4.1 times, while mitochondrial oxygen consumption rate (OCR) and ATP production decreased by 48.2% and 53.6%, respectively (*P* < 0.01), and inducible Nitric Oxide Synthase (iNOS) inhibitors could reverse the damage. Hypoglycemia induced a 5.9-fold increase in mitochondrial DNA (mtDNA) release, accompanied by a 3.8-4.2-fold upregulation in cGAS/STING protein expression (*P* < 0.01), suggesting that NO may contribute to the upregulation of the cGAS/STING pathway indirectly by promoting mtDNA release. STING knockout blocked pathway activation but did not affect mtDNA release. In the hypoglycemia group, IL-6 and TNF-α levels increased by 7.8 times and 7.6 times, respectively, cardiomyocyte survival rate dropped to 62.5%, and left ventricular function decreased by 35.3% (*P* < 0.01), all of which could be improved by STING inhibitors. Inhibition of iNOS or STING markedly restored mitochondrial function or suppressed inflammation, respectively, and combined intervention restored cardiomyocyte survival rate to 91.2% (*P* < 0.01).

**Conclusion:**

Hypoglycemia induces mitochondrial damage and mtDNA release via the iNOS-NO axis, which may subsequently promote the activation of the cGAS/STING pathway, leading to vascular and myocardial inflammatory injury. Inhibition of iNOS or STING can mitigate the damage, revealing the “NO-cGAS/STING-inflammation axis” as a core mechanism and potential therapeutic target.

## 1. Introduction

Hypoglycemia in diabetes is a common and severe complication during glycemic control, with its incidence markedly increasing with the widespread use of intensive insulin therapy. Epidemiological studies indicate that approximately 40% of patients with type 1 diabetes and 10–30% of those with type 2 diabetes experience at least one severe hypoglycemic event annually [1, 2]. Although the acute neurological symptoms of hypoglycemia are widely recognized, the chronic damage mechanisms to the cardiovascular system, especially the coronary artery endothelium, remain unclear. Clinical observations have shown that patients with recurrent hypoglycemia have an increased risk (2–3 times) of coronary artery events, independent of traditional cardiovascular risk factors [3–5]. This suggests that hypoglycemia may directly impair vascular endothelial function through specific molecular mechanisms, although the exact pathophysiological processes remain unclear. Endothelial cells, which serve as a selective barrier between blood and tissues, are a key initiating factor in atherosclerosis [6]. Recent studies showed that acute glucose fluctuations are more likely to induce endothelial oxidative stress and inflammatory responses than sustained hyperglycemia [7, 8]. Notably, reperfusion-like injury caused by hypoglycemia can lead to endothelial nitric oxide (NO) metabolic disorders, characterized by a biphasic change of initial explosive NO release and subsequent decreased bioavailability [9, 10]. This abnormal NO kinetics may activate intracellular innate immune responses through unknown pathways, becoming a new mechanism linking metabolic stress and vascular inflammation.

NO, as an important gaseous signaling molecule, plays a central role in maintaining endothelial homeostasis. Under physiological conditions, NO produced by endothelial nitric oxide synthase (eNOS) maintains vascular dilation function by activating the guanylate cyclase-cGMP pathway [11, 12]. However, under pathological conditions, excessive NO production mediated by inducible Nitric Oxide Synthase (iNOS) can lead to nitrosative stress and mitochondrial dysfunction [13]. In particular, hypoglycemia can upregulate iNOS expression by three- to five-fold through sympathetic nervous system activation and oxidative stress. This pathological increase in NO may contribute to endothelial injury through the following pathways: (1) direct inhibition of mitochondrial respiratory chain complex activity [14, 15]; (2) promotion of protein nitrosylation; (3) activation of unknown inflammatory signaling pathways [16]. Among them, whether and how NO regulates the activation of intracellular innate immune receptors is a key scientific question that remains unresolved in the field. The cyclic GMP-AMP Synthase/ Stimulator of Interferon Genes (cGAS/STING) pathway, as a cytoplasmic DNA sensing system, is traditionally considered to be mainly involved in antiviral immune responses [17]. However, recent studies have revealed that this pathway plays an important role in metabolic diseases: (1) under high-glucose conditions, mitochondrial DNA (mtDNA) escapes into the cytoplasm through defective mitophagy, activating the cGAS/STING pathway [18]; (2) the pathway promotes interferon-β (IFN-β) production through the TANK-binding Kinase 1 – Interferon Regulatory Factor 3 (TBK1-IRF3) axis; (3) genetic studies have shown that STING gene knockout can markedly improve vascular endothelial function in diabetic mice [19]. These findings strongly indicate that the pathway possibly is a “molecular bridge” linking metabolic abnormalities and vascular inflammation.

However, it should be noted that whether NO may contribute to the activation of the cGAS/STING pathway through a similar mechanism under hypoglycemic conditions remains unknown. Moreover, the unique metabolic characteristics of hypoglycemia (such as acute energy crisis and Nicotinamide Adenine Dinucleotide Phosphate (Reduced Form) (NADPH) depletion) may endow this process with a unique regulatory mode, which constitutes the core scientific question to be addressed in this article. There are currently three important gaps in the research on hypoglycemia-induced vascular injury: First, most studies focus on neuronal damage and pay insufficient attention to vascular endothelium; second, existing mechanisms are mostly concentrated on oxidative stress and calcium overload, neglecting the role of innate immunity; third, the interaction between NO and the cGAS/STING pathway under hypoglycemic conditions has not been explored. This study proposed, for the first time, the novel hypothesis that the “NO-cGAS/STING-inflammation axis” may be a core mechanism in hypoglycemia-induced endothelial injury, wherein NO potentially regulates the cGAS/STING pathway via indirect routes. As direct biochemical evidence for the S-nitrosylation of cGAS/STING by NO is not yet provided, this study aimed to elucidate the specific molecular mechanisms by which NO may modulate the cGAS/STING pathway through S-nitrosylation under hypoglycemic conditions, clarify the spatiotemporal characteristics of mtDNA release, and define the differential roles of different NO synthase isoforms (iNOS vs. eNOS) in this process. It was hoped that this study can provide a theoretical framework for understanding hypoglycemia-induced vascular complications and molecular targets for future precision interventions.

## 2. Experimental models

### 2.1. Animal experiments

#### 2.1.1. Experimental animals.

Male mice of different genotypes (with an initial number of 20 in each group) were selected, and the specific grouping and background were as follows:

The wild-type control group: 8-week-old C57BL/6J mice (purchased from Cyagen Biosciences Inc.) were used. This strain is an inbred mouse line with a stable genetic background, exhibiting no spontaneous obesity or diabetic phenotypes. At 8 weeks of age, the mice were in their youth stage, characterized by stable basal metabolism and vascular function. Age-matched with mice in other experimental groups, the influence of age differences on experimental outcomes was effectively excluded.

The diabetes model group: 8-week-old db/db mice (purchased from Changzhou Cavens Laboratory Animal Co., Ltd.) were used. This strain develops spontaneous obesity and type 2 diabetes due to a mutation in the Leptin receptor, which meets the requirements for a diabetes model in this study.

The STING knockout diabetes group: STING knockout db/db mice (with the same background strain as the db/db mice) were used to verify the role of the cGAS/STING pathway.

The iNOS knockout diabetes group: iNOS knockout db/db mice (with the same background strain as the db/db mice) were used to verify the role of NO mediated by iNOS.

Animals were randomly assigned to groups using a random number table: after one week of acclimatization feeding, all 8-week-old male mice were first stratified according to genotype (C57BL/6J, db/db, STING knockout db/db, iNOS knockout db/db) and then allocated to corresponding experimental groups within each stratum via a random number table to ensure balanced initial physiological states across groups. The randomization process was conducted by personnel not involved in experimental operations, and the grouping results were sealed until unblinding after the completion of data collection. No significant differences were observed among the groups in fasting blood glucose, heart rate, or blood lipid levels (total cholesterol, triglycerides) prior to modeling (*P* > 0.05), indicating balanced and comparable baseline characteristics and eliminating interference from variations in basic physiological states on the experimental outcomes (Table 1).

**Table 1:** Comparison of baseline physiological parameters before modeling in each group of mice.

Group	Fasting blood glucose (mmol/L)	Heart rate (beats/min)	Total cholesterol (mmol/L)	Triglycerides (mmol/L)
C57BL/6J control group	5.32 ± 0.41	586.4 ± 21.3	1.85 ± 0.23	0.62 ± 0.11
db/db Model group	5.45 ± 0.38	578.6 ± 19.7	1.92 ± 0.18	0.65 ± 0.09
STING knockout diabetic group	5.38 ± 0.45	582.1 ± 23.5	1.89 ± 0.21	0.63 ± 0.10
iNOS Knockout diabetic group	5.35 ± 0.43	584.2 ± 22.1	1.87 ± 0.19	0.61 ± 0.12
F value	0.326	0.418	0.285	0.197
*P* value	0.854	0.795	0.884	0.937

All mice were housed in an SPF environment, maintained on a 12-h light-dark cycle, and allowed free access to standard diet and water. They were provided with nesting materials and toys; they were monitored daily by professionals, and were humanely euthanized immediately if signs such as a weight loss of over 10% or lethargy appeared. The experimental protocol was reviewed and approved by the Ethics Committee of Bishan Hospital, Chongqing Medical University (Approval No.: BSKJ2024063). All animal experiments were performed in strict accordance with the Guidelines for *the Care and Use of Laboratory Animals in China*. The body weight data of mice in each group are shown in Table 2. The body weights of the db/db model group and knockout groups were significantly higher than those of the control group (*P* < 0.01), consistent with the characteristics of a diabetic obesity model.

**Table 2:** Initial average body weight of mice in each group and changes in body weight after modeling.

Group	Initial body weight	Body weight after modeling (5 days after intervention)	Change in body weight (%)
C57BL/6J control group	28.5 ± 2.1	27.8 ± 1.9	−2.46
db/db model group	52.3 ± 3.6	50.1 ± 3.2	−4.21
STING knockout diabetes group	51.8 ± 3.4	49.5 ± 2.9	−4.44
iNOS knockout diabetes group	53.1 ± 3.8	50.8 ± 3.5	−4.33

Note: Change in body weight (%) was calculated as (body weight after modeling – initial body weight)/ initial body weight × 100%. The initial body weight of the db/db model group and knockout groups was significantly higher than that of the control group (*P* < 0.01), and there was no significant difference in the change in body weight (%) among the groups (*P* > 0.05).

During surgery and invasive procedures, animals were anesthetized by intraperitoneal injection of 1% pentobarbital sodium (50 mg/kg), with the disappearance of the pedal reflex and a decrease in respiratory rate serving as indicators of anesthetic depth. After surgery, buprenorphine (0.05 mg/kg) was administered subcutaneously every 12 h for 48 h to relieve pain. At the end of the experiment, animals were euthanized in accordance with the euthanasia guidelines of the American Veterinary Medical Association. Cervical dislocation was performed under deep anesthesia (confirmed by the disappearance of corneal and pedal reflexes), and animals were euthanized immediately after the final assessment when tissue collection was required to minimize tissue degradation. Invasive procedures were performed by experienced personnel, and incisions were closed with sterile sutures and coated with antiseptic to prevent infection. This study strictly implemented blinding procedures: 1) Blinded Grouping: Animal grouping, numbering, and intervention operations were conducted by dedicated personnel. The technicians responsible for experimental assays and data analysts did not participate in the grouping process and were only aware of animal identification numbers, with no knowledge of group assignments. 2) Blinded Assessment: During procedures such as Western blot development, immunofluorescence image acquisition, transmission electron microscopy observation, and hemodynamic parameter recording, operators handled samples based solely on identification numbers and were not informed of the corresponding group assignments. 3) Blinded Statistical Analysis: After data compilation, blinding was lifted by a third-party statistician before statistical analysis was conducted to prevent subjective bias.

#### 2.1.2. Construction of diabetic hypoglycemia model.

Diabetes-modeling screening: 8-week-old db/db mice with fasting blood glucose ≥11.1 mmol/L for 3 consecutive days were confirmed to have successful diabetes modeling; 8-week-old C57BL/6J mice served as a normal control, with consistent age with diabetic model groups.

Hypoglycemia induction: All model groups (including the db/db group, STING knockout group, and iNOS knockout group) were subjected to the following treatment: After fasting for 12 h (with free access to water), intraperitoneal injection of Novolin R insulin (15 IU/kg, batch number J20050019, Novo Nordisk, Denmark) was administered. Tail-vein blood glucose was measured every 20 min, and when blood glucose levels dropped to ≤3.5 mmol/L, hypoglycemia induction was deemed successful. This state was maintained for 1 h, followed by intraperitoneal injection of 20% glucose PBS solution (1 g/kg, R21823-500, Shanghai Shangbao Biotechnology Co., Ltd.) to restore blood glucose levels [20]. This procedure was performed once daily for five consecutive days. The control group (C57BL/6J mice) received injection of an equivalent volume of normal saline without inducing hypoglycemia.

During the hypoglycemia induction process, the insulin injection speed (0.1 mL/min) and the timing of glucose solution resuscitation were strictly controlled to ensure that the duration of hypoglycemia (1 h) and the rate of blood glucose recovery (blood glucose restored to ≥7.0 mmol/L within 30 min after resuscitation) remained consistent across all mouse groups. The modeling was conducted daily at a fixed time between 9:00 AM and 11:00 AM to minimize the influence of circadian rhythms on the experimental results.

#### 2.1.3. Modeling outcomes.

Initially, there were 20 mice in each group, and the final survival and successful modeling situations were as follows:

In C57 control group, 18 mice survived (2 died of natural aging);

In db/db model group, 17 mice were successfully modeled (3 were excluded due to severe hyperglycemic complications);

In STING knockout group, 19 mice were successfully modeled (1 was excluded due to failed genotype identification);

In iNOS knockout group, 18 mice were successfully modeled (2 were excluded due to poor tolerance to hypoglycemia).

### 2.2. Cell model

#### 2.2.1. Primary cell isolation and culture.

a Primary mouse coronary artery endothelial cells (used for hypoglycemia model research):

The time point for isolation was 24 h after the completion of the hypoglycemia model construction (that is, 24 h after the end of 5 consecutive days of hypoglycemia induction), and the cells were isolated from the hearts of 6 db/db diabetic mice (8 ± 2 weeks old). The specific steps were as follows:

The hearts were removed from the heparinized (1 U/g) and anesthetized mice and washed in 4°C pre-cooled Krebs-Ringer buffer (containing Ca² ⁺ /Mg² ⁺ , T10489, Shanghai Shangbao Biotech Co., Ltd.); the anterior wall of the left ventricle was incised, and the left/right atria, interventricular septum, epicardium, and the outer 1/4 of the left ventricle were removed, leaving the coronary artery-enriched area; the tissue was minced and added to 0.2% Type II collagenase (Worthington) pre-warmed at 37°C, and digested in a 37°C water bath for 15−30 min; 0.05% trypsin + 1% EDTA (Gibco) was added to continue digestion for 30 min; the digest was filtered through a 100 μm sieve and centrifuged at 100 × g for 10 min; digestion was terminated by adding 10% fetal bovine serum (Nanjing Saihongrui Biotechnology Co., Ltd.), and after a second centrifugation, the precipitate was resuspended in endothelial cell culture medium (EGM-2, ScienCell); during the first passage, positive selection with anti-mouse CD31 microbeads was used, and the purity was verified by flow cytometry to be > 90%.

Cultivation conditions: EGM-2 medium (containing 2% FBS, VEGF, EGF, FGF) was used, and the cells were cultured in an environment of 37°C and 5% CO₂, with medium change every 2 days.

b Primary mouse cardiomyocytes (used for myocardial injury research):

From C57BL/6J mice (without hypoglycemia induction), the steps were as follows:

After the heart was minced, it was placed in a petri dish containing 0.075% trypsin (Gibco) and digested at 4°C for 24 h (to inhibit fibroblast proliferation); the digested tissue was then transferred to 0.05% Type II collagenase pre-warmed at 37°C, and digested by magnetic stirring at 120 rpm and 34–35°C for 30 min until the viscous material disappeared; DMEM-F12 medium (T10299, Shanghai Shangbao Biotech Co., Ltd.) containing 10% FBS was added, and the mixture was centrifuged at 1000 rpm for 10 min; the precipitate was resuspended in DMEM-F12 medium containing 15% FBS and Brdu (67 μL/20 mL), and filtered through a 200-mesh sieve.

The culture conditions were as follows: DMEM-F12 + 15% FBS + Brdu was used, and the culture dishes had been pre-coated with 10 μg/mL laminin to promote adhesion.

#### 2.2.2. Construction of hypoglycemia cell model.

a Experimental groups:

The high glucose group (HG) was prepared with DMEM containing 30 mM glucose (4.5 g/L basic DMEM plus 1 M glucose solution).

The low glucose group (LG) was prepared with sugar-free DMEM containing 1 mM glucose (sugar-free DMEM plus 1 M glucose solution to adjust the concentration).

b The model was established as follows:

The coronary artery endothelial cells were cultured in high-glucose medium (30 mM) for 48 h (to simulate the diabetic environment), then treated with low-glucose medium (1 mM) for 2 h, and subsequently recultured in high-glucose medium (30 mM) for 6 h. This procedure was repeated (three times), with a total duration of 24 h. The duration of low-glucose treatment was ≤ 2 h to avoid irreversible cell damage. Three replicate wells were set for each group, and the experiment was performed in triplicate [21]. During alternating high and low glucose treatments, the medium replacement volume was precisely controlled using an accurate pipette (2 mL per well). Uniform cell culture conditions across all groups were ensured by real-time monitoring of the CO₂ concentration (5%) and temperature (37°C) inside the incubator. The duration of low glucose treatment (1 mM) was strictly limited to 2 hours, which was verified through preliminary experiments to be sufficient for inducing cellular stress responses without causing irreversible damage.

**Fig 1:**
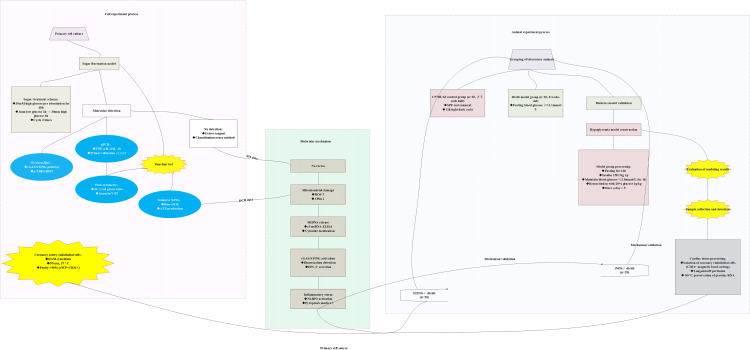
Flowchart of the technical route.

The technical route of this study is illustrated in Fig 1.

## 3. Detection indicators and methods

### 3.1. Detection of cGAS/STING pathway activation and inflammatory response

#### 3.1.1. Subcellular localization detection (immunofluorescence).

Cardiomyocytes/endothelial cells were seeded on poly-L-lysine-coated coverslips at 5 × 10^4^ per well (24 h). They were fixed with 4% paraformaldehyde (PFA) (Catalog number 30525-89-4, Jiangsu Wenru Technology Chemical Co., Ltd.) (25°C, 20 min), followed by three washes with PBS (3 min each). Samples were treated with 0.1% Triton X-100 (in PBS) (15 min) to achieve permeabilization, followed by three PBS rinses. Blocking was carried out adopting 5% BSA (0.1% Triton X-100) [Yishijiu (Lianyungang, Jiangsu) Biotechnology Co., Ltd.. (25°C, 1 h). Overnight incubation at 4°C was performed with first antibodies (Ab), including cGAS (Catalog number YSH605050, Shanghai Yishenghe Biotechnology Co., Ltd., 1:200), STING (Shanghai Yuanye Biotechnology Co., Ltd., 1:200), and mitochondrial marker (Catalog number CS22668, AAT Bioquest, 1:500). Secondary Ab were applied (37°C, 1 h) (light-protected), including Alexa Fluor 488/594-conjugated secondary Ab (Thermo Fisher Scientific China, 1:500). The nuclei were stained with DAPI (5 μg/mL) (Shanghai Shangbao Biotechnology Co., Ltd.) (5 min), followed by washing with PBS and mounting with an anti-fade reagent (ProLong Gold) [Yishijiu (Lianyungang, Jiangsu) Biotechnology Co., Ltd... Sample visualization was carried out under a confocal microscope (Catalog number SZ61TR, Leica). Five fields of view were randomly selected from each group, and the colocalization coefficient (Pearson’s coefficient) of cGAS/STING with the mitochondrial marker was calculated by Image J software. The experiment was independently repeated three times. Nuclear fluorescence intensity of NF-κB p65 was quantified using ImageJ software, and the relative expression was normalized to the control group.

#### 3.1.2. Protein expression detection.

Following lysis in RIPA buffer containing protease inhibitors, quantification was carried out by BCA assay. 30 μg of protein was loaded for electrophoretic separation, followed by wet transfer to a PVDF membrane (100 V, 90 min). Overnight incubation (4°C) was carried out with first Ab, including cGAS (1:1000), STING (1:1000). Secondary Ab were applied (25°C, 1 h), including HRP-conjugated anti-rabbit/mouse IgG (1:5000) (Catalog number C2210, APPLYGEN). The proteins were visualized using ECL, and the gray values were determined with Image Lab software, with β-actin serving as the control for normalization [22]. The experiment was independently repeated three times, and the results were expressed as mean ± standard deviation ($\overset{―}{\text{x}}$±s).

The quality control standards for cGAS/STING protein detection were as follows: the protein sample concentration was required to be ≥ 1 mg/mL; SDS-PAGE electrophoresis bands needed to be clear and without trailing; transfer efficiency was verified using pre-stained protein markers (with complete transfer of bands in the 25–150 kDa range); and the coefficient of variation for β-actin internal reference grayscale values was required to be ≤ 10% to ensure uniform protein loading.

#### 3.1.3. Inflammatory factor detection.

ELISA for secreted proteins: The supernatant was collected after centrifugation (2,000 rpm, 10 min) and stored at −80°C. Overnight incubation (4°C) was carried out for coating Ab (IL-1β/IL-6/IL-8/ICAM1/GMCSF/IFN-β, Thermo Fisher Scientific China). Subsequently, samples/standards (50 μL per well) were applied (37°C, 2 h). The HRP-conjugated secondary Ab were applied (1 h), followed by TMB color development and measurement of the OD value at 450 nm. The concentrations of each factor (pg/mL) were calculated according to the standard curve, and the experiment was independently repeated three times.

qPCR for mRNA levels: Total RNA extraction was conducted adopting the Trizol approach, and the concentration was measured with a Nanodrop (A260/280 ≥ 1.8). cDNA was synthesized using the PrimeScript RT kit (Catalog number RR064A, Takara). The primer sequences are shown in Table 3, and the experiment was independently repeated three times.

**Table 3:** qPCR primer sequences and reaction conditions.

Item	Parameter/sequence
RNA extraction	Trizol method
RNA quality assessment	Nanodrop measurement (A260/280 ≥ 1.8)
cDNA synthesis	PrimeScript RT kit (37°C 15 min, 85°C 5 s)
qPCR protocol	95°C for 30 s → 40 cycles (95°C for 5 s, 60°C for 30 s) → Melting curve analysis
Data analysis method	2 ⁻ ΔΔCt method
Primer sequences (5’ → 3’)	IL-1β: F-GCAACTGTTCCTGAACTCA; R-CTCGGAGCCTGTAGTGCAG
	IL-6: F-ACAAGTCCGGAGAGGAGAC; R-TTGGATGGTCTTGGTCCTTAG
	TNF-α: F-CCACCACGCTCTTCTGTCT; R-GGTTGTCTTTGAGATCCATGCC
	NF-κB p65: F-GGAGACCTTCCTGATGGACA; R-GCTGGGGTCATTTTCACGTA
	GAPDH: F-AGGTCGGTGTGAACGGATTTG; R-TGTAGACCATGTAGTTGAGGTCA

### 3.2. Detection of NO and mitochondrial damage

#### 3.2.1. NO detection.

a Detection of NO release by electrochemical method

Cells were seeded in a 6-well plate and cultured until reaching 80% confluence. The medium was then replaced with phenol red-free medium (Catalog number 11835030, Thermo Fisher Scientific, China) and incubation was performed at 37°C for 1 h. The medium was collected, and centrifugation was carried out at 4°C and 3000 rpm for 10 min, and the supernatant was reserved for later use. The ISO-NOP NO sensor (World Precision Instruments) was calibrated with a sodium nitrite standard curve ranging from 0.1 μM to 10 μM (R² ≥ 0.99). The samples were placed in a constant temperature bath at 37°C, and a 2 mm diameter NO electrode was inserted. After recording a stable baseline, L-arginine (1 mM, Catalog number 5256-76-8, Tai’an Heguang Fine Chemical Co., Ltd.) was added to stimulate NO release, and the peak changes within 5 min were continuously monitored. The NO concentration (nM/mg protein) was calculated by LabTrax 4.0 software, and the experiment was independently repeated three times.

b Detection of NO metabolites by ELISA

The cell lysate or serum samples were added to Griess reagent (Yishijiu (Lianyungang, Jiangsu) Biotechnology Co., Ltd.) at a ratio of 1:1 and reacted in the dark at room temperature for 10 min. In a 96-well plate, standards (0–100 μM sodium nitrite), samples, and chromogen A + B (50 μL each) were added successively, and the mixture was reacted in the dark at 25°C for 10 min. The OD value at 540 nm was measured using a microplate reader. The NO concentration (μM) was calculated according to the standard curve, and the experiment was independently repeated three times.

Quality control for NO release detection by the electrochemical method: The sensor calibration correlation coefficient was R² ≥ 0.995. The sample equilibration time before detection was ≥ 30 minutes. Each sample was tested in triplicate, and the mean of the peak values was taken as the final result. If the coefficient of variation among the three replicate measurements exceeded 15%, the sample was reanalyzed.

#### 3.2.2. Mitochondrial function detection.

a Mitochondrial oxygen consumption rate (OCR) and ATP production

Cardiomyocytes were seeded at a density of 8 × 10⁴ cells per well in the XF96 cell culture plate and cultured for 24 h at 37°C. The XF base medium (containing 10 mM glucose, 1 mM sodium pyruvate, 2 mM GlutaMAX, Catalog number 103575−100, Thermo Fisher Scientific, China) was used, and the cells were incubated at 37°C without CO₂ for 1 h. The XF96 Seahorse Analyzer was used for detection: Baseline measurement was performed three times → Oligomycin (1 μM, Catalog number O4876-5 mg, Sigma) was injected → Measurement was performed three times → FCCP (0.5 μM, Catalog number D1975, Sigma) was injected → Measurement was performed three times → Rotenone/Antimycin A (0.5 μM, Catalog Number DE0044/ S53076-1 mg, Lemeitan/Shanghai Yuanye Biotechnology Co., Ltd.) was injected → Measurement was performed three times. The Wave software was used for calculation: Basal respiration = Baseline OCR – OCR after rotenone; ATP production = OCR before oligomycin – OCR after oligomycin; Maximal respiration = OCR after FCCP – OCR after rotenone. Six replicate wells were set for each group, and the experiment was independently repeated three times.

b Activity of respiratory chain complexes

Western blot detection was as follows: The abovementioned method was used. The primary antibodies were Complex I (NDUFB8, Catalog number ab251160, Abcam, 1:1000), Complex II (SDHB, Catalog number ZY756Mu015, HZbscience, 1:1000), and Complex V (ATP5A, Catalog number PA2000−1517, Aipudi, 1:1000). β-actin was used as the internal reference, and the relative expression levels were calculated.

The Clark oxygen electrode method was employed. Myocardial tissue homogenate was centrifuged at 600 g for 10 min (4°C) to obtain the supernatant, which was then centrifuged at 10000 g for 15 min (4°C) to obtain the mitochondrial pellet. The reaction system (1 mL) consisted of respiration buffer (225 mM mannitol, 75 mM sucrose, 10 mM KCl), 5 mM glutamate (substrate), and 0.5 mg mitochondrial protein. After incubation at 37°C for 5 min, 200 μM ADP was added to initiate state 3 respiration, and the OCR was recorded. State 4 respiration was defined as the OCR after ADP depletion. The respiratory control ratio (RCR) was calculated as state 3 oxygen consumption divided by state 4 oxygen consumption. The experiment was independently repeated three times.

#### 3.2.3. Mitochondrial structural detection.

a Observation by transmission electron microscope

Myocardial tissue (1 mm³) was taken and fixed in 2.5% glutaraldehyde (0.1 M PBS, pH 7.4) at 4°C for 24 h, followed by postfixation in 1% osmium tetroxide for 2 h. It was then dehydrated through an ethanol gradient (50% → 70% → 90% → 100%), embedded in epoxy resin (Epon 812), and polymerized at 60°C for 48 h. Ultrathin sections (70 nm) were double-stained with uranyl acetate and lead citrate and observed under a transmission electron microscope (model Tecnai G2 Spirit BioTWIN, FEI) at 20000 × magnification. Ten fields of view were randomly selected for each group and scored according to the Flameng criteria: 0 points (intact structure), 1 point (mild swelling, partial cristae disruption), 2 points (marked vacuolization), 3 points (complete loss of cristae). The results were expressed as the mean score.

b Detection of mitochondrial membrane potential

Cells were seeded into a confocal dish and incubated with JC-1 staining solution (5 μg/mL, Catalog number C2006, Shanghai Beyotime Biotechnology Co., Ltd.) at 37°C for 20 min, followed by three washes with PBS. Observation under a fluorescence microscope (model IX73, Olympus): Normal mitochondrial membrane potential appeared as red fluorescence (Ex/Em = 585/590 nm), while depolarization appeared as green fluorescence (Ex/Em = 514/529 nm). Detection by flow cytometer (model Accuri C6, Thermo Fisher Scientific), the red/green fluorescence intensity ratio was calculated. Three replicate wells were set for each group, and the experiment was independently repeated three times.

c Detection of mitochondrial permeability transition pore (mPTP) opening

Cells were incubated with Calcein-AM (1 μM) and CoCl₂ (1 mM) at 37°C for 30 min (CoCl₂ quenched cytoplasmic fluorescence), and mitochondrial Calcein fluorescence (Ex/Em = 488/515 nm) was observed under a confocal microscope (model LSM880, Zeiss). A decrease in fluorescence intensity indicated mPTP opening. Five cells were randomly selected from each group, and the fluorescence intensity was quantitatively analyzed by *Image J*. The experiment was independently repeated three times.

### 3.3. Detection of cardiomyocyte/heart injury

#### 3.3.1. Detection at the cellular level.

Cardiomyocyte viability: Detection was performed adopting the WST-1/Cell Viability kit. Primary cardiomyocytes in a 96-well plate (1 × 10^4^ per well) were cultured (24 h). The medium was exchanged, WST-1 reagent (10 μL/well, Shanghai Shangbao Biotechnology Co., Ltd.) was applied, and incubation (light-protected) (37°C, 2 h) was carried out. The OD value at 450 nm (reference 630 nm) was determined. The viability calculation equation is as follows:


$$\begin{array}{c} CellularActivity\left(\%\right)=\frac{ExperimentalGroupOD-BlankGroupOD}{ControlGroupOD-BlankGroupOD}\times 100\% \end{array}$$ (1)

Control was set as follows: Blank group was treated with cell-free culture medium + WST-1; Positive control was treated with 0.1% Triton X-100 (100% cytotoxicity). Six replicate wells were set for each group, and the experiment was independently repeated three times.

Cardiomyocyte function was assessed. Cardiomyocytes were seeded on laminin-coated glass-bottom culture dishes (35 mm) and cultured for 48 h until synchronized contraction was achieved. The IonOptix system was preheated, and the sampling frequency (240 Hz) and electrical stimulation parameters (1 Hz, 5 ms pulse width) were set. Contraction function was assessed, with indicators including contraction amplitude (μm), shortening fraction (%) = (diastolic length – systolic length)/ diastolic length × 100, and maximum contraction/relaxation rates (±dL/dt, μm/s). The IonWizard software was adopted to automatically track cell edge movement, and the average of 10 consecutive cycles was computed. Five cells were examined for each group, and the experiment was independently repeated three times.

#### 3.3.2. Organ-level detection (Langendorff perfusion system).

Mice were heparinized (1 U/g) and then anesthetized. The thorax was opened, and the heart was rapidly excised and placed in 4°C Krebs-Henseleit buffer [Yishijiu (Lianyungang, Jiangsu) Biotechnology Co., Ltd.. (KHB, containing 118 mM NaCl, 4.7 mM KCl, 1.2 mM MgSO₄, 1.2 mM KH₂PO₄, 25 mM NaHCO₃, 11 mM glucose, 2.5 mM CaCl₂, pH 7.4). A perfusion cannula was inserted into the aortic arch, and the heart was perfused with oxygenated KHB at a constant pressure of 80 mmHg and 37°C (95% O₂/5% CO₂). Functional parameters were then monitored by inserting a pressure transducer (AD Instruments) into the left ventricular apex. The indicators measured included left ventricular systolic pressure (LVSP, mmHg), left ventricular end-diastolic pressure (LVEDP, mmHg), left ventricular developed pressure (LVDP = LVSP – LVEDP), and maximum contraction/relaxation rates (±dp/dt_max_, mmHg/s). Baseline data were recorded after stable perfusion for 20 min. The heart was then perfused with glucose-free KHB + insulin (15 mU/mL) (30 min), returning to normal KHB. The changes in each parameter compared to baseline were computed adopting LabChart software. Six mice were assigned to each group.

### 3.4. Statistical analysis

All experimental data were expressed as mean ± standard deviation ($\overset{―}{\text{x}}$±s) and analyzed using *SPSS 27.0*. One-way analysis of variance (ANOVA) was used for comparisons among multiple groups, and LSD-*t* test was applied for pairwise comparisons between groups; independen*t* samples *t*-test was used for comparisons between *t*wo groups. *P* < 0.05 was considered statistically significant.

## 4. Results

### 4.1. Elevated NO levels and mitochondrial damage in the context of diabetic hypoglycemia

**Fig 2:**
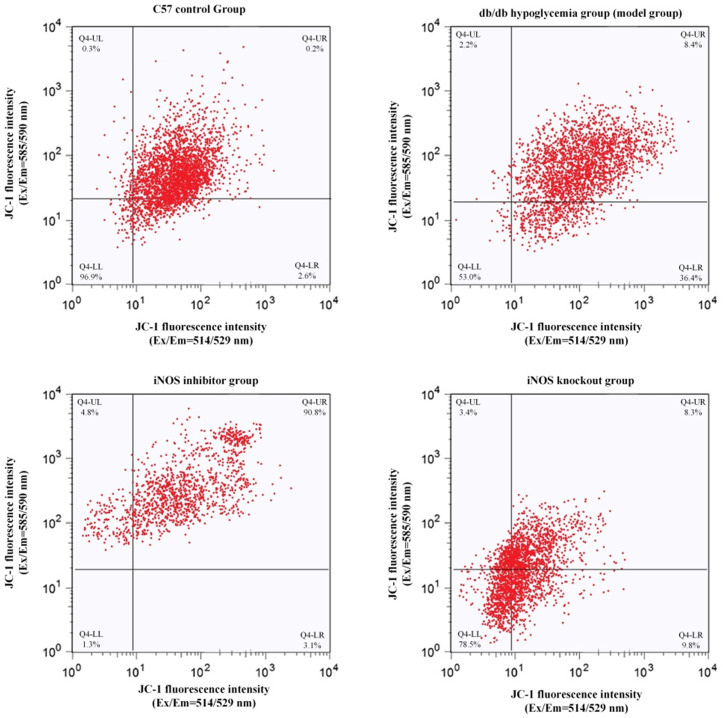
Flow cytometry map.

**Fig 3:**
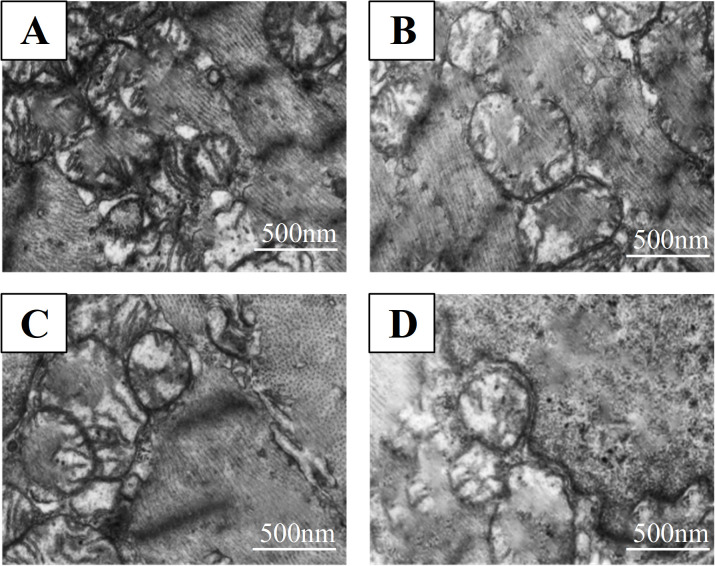
Transmission electron microscope images of mitochondrial structure (20000×). Note: A: C57 control group; B: db/db hypoglycemia group (model group); C: iNOS knockout group; D: iNOS inhibitor group.

The results showed that in the db/db hypoglycemia group, the NO level was increased by 4.1 times compared with the C57 control group (*P* < 0.01), accompanied by significant mitochondrial dysfunction (OCR decreased by 48.2%, ATP decreased by 53.6%, both *P* < 0.01). iNOS knockout or inhibitor treatment significantly reversed these changes (*e.g.,* OCR was restored to 228−235 pmol/min, ATP was restored to 9.5–9.7 nmol/mg protein, both *P* < 0.01), and there was no difference between the two groups (*P* > 0.05) (Table 4). Flow cytometry (JC-1 staining) showed a significant increase in the proportion of mitochondrial depolarization in the model group, which was normalized after iNOS intervention (Fig 2). Transmission electron microscope showed that the model group had mitochondrial cristae disruption and vacuolization (Flameng score 2.8 ± 0.4), and the structural damage was alleviated in the iNOS knockout group (Fig 3).

**Table 4:** Comparison of NO levels and mitochondrial function indicators.

Group	NO (μM)	OCR (pmol/min)	ATP (nmol/mg protein)	Membrane potential (red/green ratio)	mPTP opening rate (%)
C57 control group	2.1 ± 0.3	280 ± 25	12.5 ± 1.2	5.2 ± 0.8	8.3 ± 2.1
db/db hypoglycemia group (model group)	8.7 ± 1.1**	145 ± 18**	5.8 ± 0.9**	1.8 ± 0.5**	42.6 ± 6.3**
iNOS inhibitor group (10 μM)	3.5 ± 0.6##	235 ± 22##	9.7 ± 1.1##	4.1 ± 0.7##	15.2 ± 3.4##
iNOS knockout group (iNOS⁻/⁻ db/db)	3.2 ± 0.5##	228 ± 20##	9.5 ± 1.0##	3.9 ± 0.6##	16.1 ± 3.2##

Note: Compared with the C57 control group, **P < 0.01 (F = 24.63, F = 31.27, F = 28.59, F = 35.12, F = 29.84); compared with the db/db hypoglycemia group, ##*P* < 0.01 (F = 18.45, F = 22.31, F = 20.76, F = 25.38, F = 21.65)

The results directly confirmed that NO overproduction mediated by iNOS is the core driving force of mitochondrial damage. The equivalent effects of iNOS knockout and inhibitors ruled out the interference of other subtypes such as eNOS and clarified the source of NO. The significant decline in mitochondrial functional indicators (OCR, ATP) in the model group was directly associated with the elevated NO levels [23], and the improvement in mitochondrial structure and function after iNOS knockout (Fig 3) provided key evidence for the theory that iNOS-NO causes mitochondrial damage. Compared with the results showing that STING knockout had no effect on mitochondrial structure (Table 3 in Section 4.2), it could be concluded that mitochondrial damage was the result of NO’s independent action and was unrelated to downstream pathways. Although the iNOS inhibitor partially improved mitochondrial function (ATP recovery to 78.5%), it failed to completely reverse the damage [24], suggesting that in addition to NO, other damaging factors (such as oxidative stress) might be involved in a synergistic effect [25]. The mitochondrial structural damage shown by transmission electron microscope (cristae disruption and vacuolization) [26] further supported the mechanism that NO caused functional impairment by disrupting mitochondrial integrity, and the increased opening rate of mPTP was a direct functional manifestation of the structural damage.

**Fig 4:**
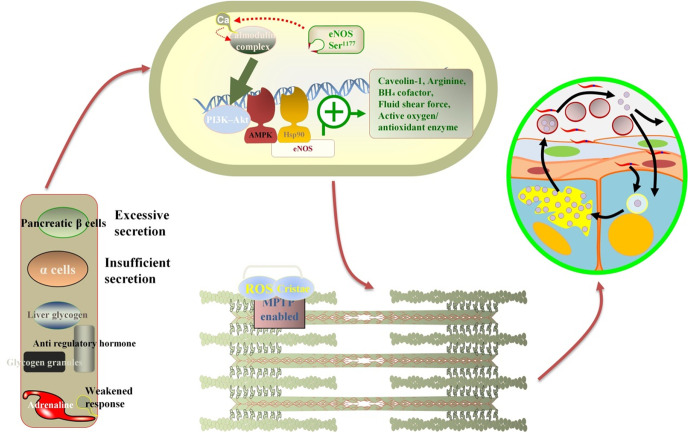
Schematic diagram of endothelial injury mechanism triggered by diabetic hypoglycemia.

Fig 4 integrates the initiating mechanisms of diabetic hypoglycemia → NO → mitochondrial damage, with the core pathway being blood glucose fluctuation → excessive NO production in vascular endothelial cells → mitochondrial structural damage (cristae disruption/vacuolization) → impaired energy metabolism (decreased ATP synthesis).

### 4.2. mtDNA release to activate the cGAS/STING pathway

**Fig 5:**
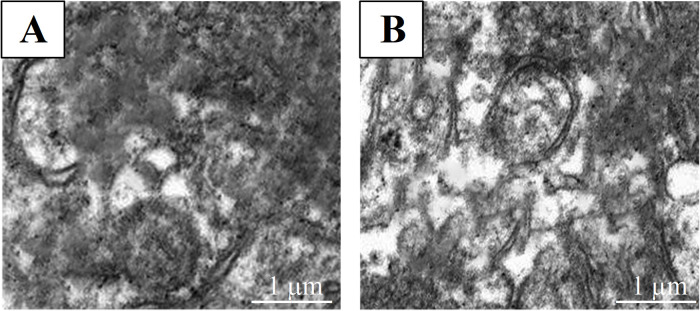
Transmission electron microscope images of mitochondrial structure (20000×). Note: A: db/db hypoglycemia group (model group); B: STING knockout group.

**Fig 6:**
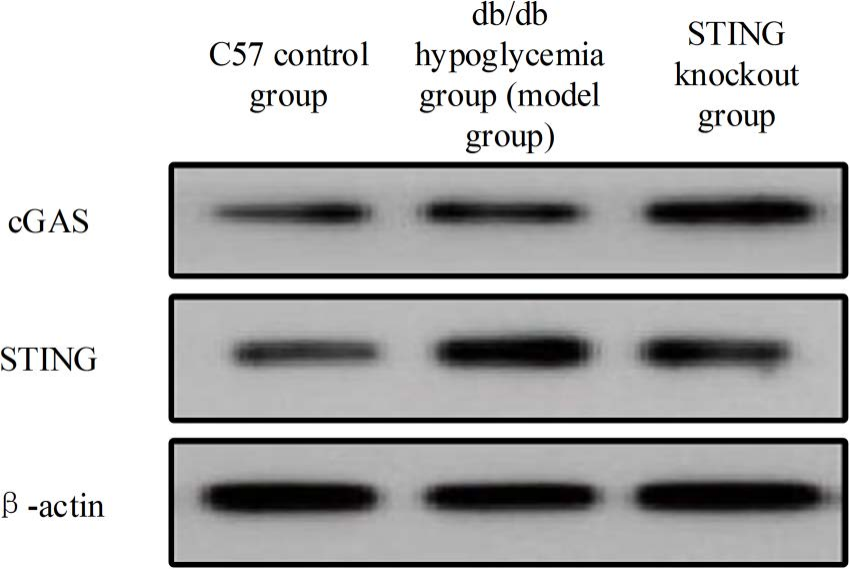
Western blot bands.

The results indicated that in the model group, the release of mtDNA was increased by 5.9 times (*P* < 0.01), the expression of cGAS/STING proteins was upregulated by 3.8–4.2 times, and IFN-β was elevated by 6.3 times (*P* < 0.01). STING knockout could block the pathway activation (STING expression decreased to 0.2 ± 0.1), but it did not affect the release of mtDNA (905 ± 138 copies/μg) and mitochondrial structure (Flameng score 2.6 ± 0.3, *P* > 0.05) (Table 5). Transmission electron microscope showed that the mitochondria in the STING knockout group still had cristae disruption (Fig 5), and Western blot confirmed that the cGAS/STING bands were enhanced in the model group, while the STING band almost disappeared in the STING knockout group (Fig 6).

**Table 5:** Comparison of Flameng scores for mitochondrial structural damage.

Group	Flameng score	mtDNA (copies/μg)	cGAS (relative expression)	STING (relative expression)	IFN-β (pg/mL)
C57 control group	0.2 ± 0.1	152 ± 35	1.0 ± 0.2	1.0 ± 0.3	25.6 ± 6.3
db/db hypoglycemia group (model group)	2.8 ± 0.4**	892 ± 124**	3.8 ± 0.6**	4.2 ± 0.7**	186.5 ± 32.4**
STING knockout group	2.6 ± 0.3	905 ± 138	3.7 ± 0.5**	0.2 ± 0.1##	41.2 ± 9.7##

Note: Compared with the C57 control group, **P < 0.01 (F = 42.36, F = 38.72, F = 35.49, F = 40.15, F = 33.67); compared with the model group, ##*P* < 0.01 (F = 28.53, F = 0.12, F = 0.31, F = 56.82, F = 45.29)

The result that STING knockout had no effect on mitochondrial structure (Table 3) further supported the conclusion that mitochondrial damage is an independent effect of NO and is unrelated to pathway activation. The significant reduction in mtDNA release in the iNOS knockout group, in contrast to the model group, confirming that NO is a key regulator of mtDNA release. As a ligand of cGAS, the amount of mtDNA released is positively correlated with the degree of cGAS/STING activation [27], suggesting that NO initiates the pathway through a cascade reaction of mtDNA release caused by mitochondrial damage. Although STING knockout can completely block pathway activation (STING expression decreased to 0.2 ± 0.1) [28], it does not affect mtDNA release, clarifying that the pathway is downstream of mtDNA [29]. IFN-β, as a classic downstream factor of the pathway, has elevated levels (186.5 ± 32.4 pg/mL), indicating that the innate immune response is activated [30], which may exacerbate endothelial damage by recruiting immune cells and provide a trigger for subsequent inflammatory responses [31].

### 4.3. Inflammatory cytokine release and cardiomyocyte injury

**Fig 7:**
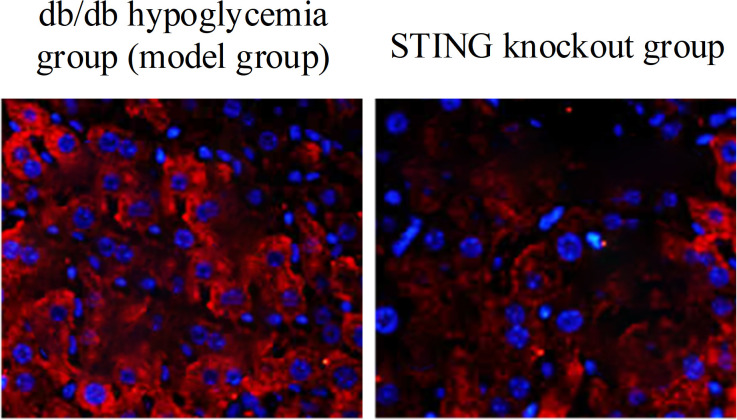
Immunofluorescence of NF-κB p65 nuclear translocation (200×).

The results showed that in the model group, IL-6 and TNF-α were increased by 7.8 times and 7.6 times, respectively, IFN-β was elevated by 6.3 times, and NF-κB p65 nuclear fluorescence intensity, IL-1β, etc. were also significantly upregulated (*P* < 0.01). The STING inhibitor could reduce IL-6 by 65.5%, TNF-α by 65.3%, and IFN-β by 77.9% (Table 6). Immunofluorescence showed an increase in NF-κB p65 nuclear translocation in the model group, and the nuclear fluorescence intensity was quantified by ImageJ (Fig 7).

**Table 6:** Levels of inflammatory cytokines and cardiac function indicators.

Indicator	C57 control group	db/db hypoglycemia group (model group)	STING inhibitor group
NF-κB p65 (nuclear fluorescence intensity, relative expression)	1.0 ± 0.2	3.2 ± 0.5**	1.4 ± 0.3##
IL-1β (pg/mL)	1.8 ± 0.4	12.3 ± 1.5**	4.2 ± 0.8##
IL-8 (pg/mL)	12.6 ± 2.1	96.5 ± 10.2**	35.7 ± 4.2##
ICAM-1 (ng/mL)	15.2 ± 3.4	89.7 ± 9.3**	32.5 ± 5.1##
GM-CSF (pg/mL)	6.3 ± 1.2	45.6 ± 5.8**	16.8 ± 2.3##
IL-6 (pg/mL)	32.5 ± 8.2	286.4 ± 45.3**	98.7 ± 21.6##
TNF-α (pg/mL)	28.7 ± 6.5	245.8 ± 39.1**	85.3 ± 18.4##

Note: Compared with the C57 control group, **P < 0.01 (F = 37.29, F = 41.56, F = 39.82, F = 43.17, F = 36.54, F = 38.67, F = 35.21); compared with the model group, ##*P* < 0.01 (F = 29.34, F = 33.78, F = 31.25, F = 36.89, F = 28.43, F = 32.15, F = 30.76)

These inflammatory factors have clear roles in coronary artery endothelial damage. IFN-β, as a direct downstream factor of cGAS/STING, exacerbates vascular wall inflammatory infiltration by recruiting monocytes through the induction of chemokines (such as CXCL10) [32]; IL-6 disrupts endothelial barrier integrity and increases vascular permeability by activating the STAT3 pathway, promoting the extravasation of inflammatory factors [33]; TNF-α directly induces endothelial cell apoptosis and upregulates adhesion molecules (such as ICAM-1), facilitating leukocyte adhesion and migration [34]. The high expression of IL-6 and TNF-α in the model group was directly associated with decreased cardiomyocyte survival and reduced LVDP, indicating that inflammatory factors are key mediators linking vascular endothelial damage to myocardial dysfunction. The STING inhibitor had the strongest inhibitory effect on IFN-β (77.9%), but a weaker effect on TNF-α. IFN-β mainly depends on the cGAS/STING pathway, while TNF-α may be partially produced through other pathways (such as TLR4), and this heterogeneity provides a basis for precise anti-inflammatory strategies [35].

**Fig 8:**
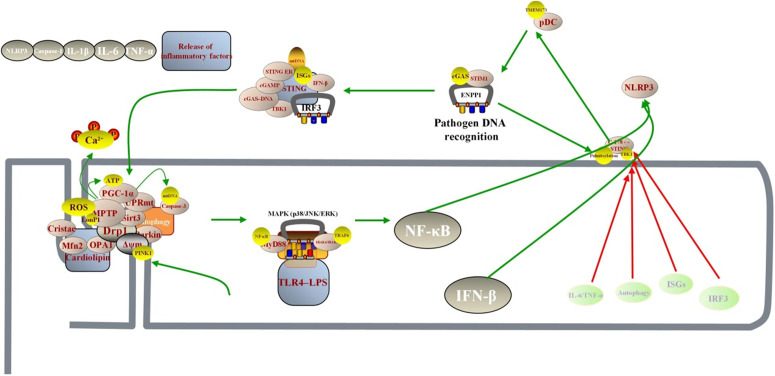
Schematic diagram of inflammation storm mechanism triggered by mitochondrial damage.

Fig 8 illustrates the cascade of events following injury, with the core pathway being damaged mitochondria releasing mtDNA → activating the cGAS/STING pathway → promoting the release of IL-6/TNF-α → leading to cardiomyocyte death.

### 4.4. Protective effects of interventions

**Fig 9:**
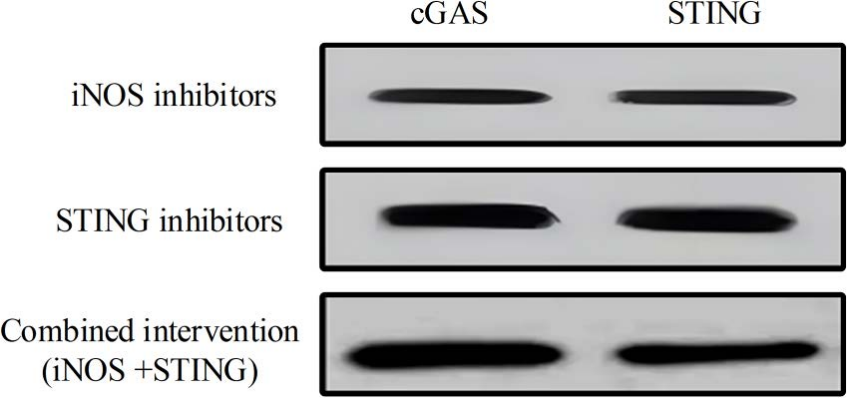
Western blot analysis of the combined intervention’s impact on the cGAS/STING pathway.

The results indicated that the iNOS inhibitor restored OCR by 81.3%, the STING inhibitor reduced IL-6 by 65.5%, and the combined intervention restored cardiomyocyte survival to 91.2% (Table 5). Western blot confirmed that the combined intervention could normalize the expression of cGAS/STING (Fig 9). The significant improvement in mitochondrial function by the iNOS inhibitor (Table 7) corroborated that NO is a core target of mitochondrial damage; the potent suppression of inflammatory factors by the STING inhibitor demonstrated that the pathway is a key amplifier of inflammation. The synergistic effect of the combined intervention essentially involves simultaneously blocking NO-mediated mitochondrial damage and cGAS/STING-mediated inflammatory burst. The iNOS inhibitor reduced NO production, thereby decreasing mitochondrial damage and mtDNA release at the source; the STING inhibitor blocked downstream inflammatory signaling, and the two interventions were complementary. It is particularly noteworthy that the combined intervention restored cardiomyocyte survival to 91.2%, which was much higher than that achieved by either intervention alone, confirming the combined effect of the NO-cGAS/STING pathway and the inflammatory axis and providing experimental evidence for clinical combined target therapy.

**Table 7:** Comparison of improvement rates of key indicators.

Intervention measure	OCR recovery rate (%)	ATP recovery rate (%)	IL-6 reduction rate (%)	Cell survival rate increase (%)	LVDP improvement rate (%)
iNOS inhibitor	81.3 ± 7.2**	78.5 ± 6.8**	52.4 ± 8.1	68.2 ± 9.3**	55.7 ± 7.6*
STING inhibitor	62.4 ± 6.5*	59.8 ± 5.9*	65.5 ± 7.8**	74.6 ± 8.4**	63.2 ± 6.9**
Combined intervention (iNOS + STING)	89.7 ± 8.1**	85.2 ± 7.3**	71.8 ± 6.9**	91.2 ± 10.5**	82.4 ± 9.1**

Note: Compared with the model group, *P < 0.05, **P < 0.01 (F = 22.58, F = 20.43, F = 25.76, F = 28.31); Improvement rate = (Intervention group value – Model group value)/ (Control group value – Model group value) ×100%

## 5. Conclusion

This study confirmed that diabetic hypoglycemia induces mitochondrial damage and mtDNA release through NO overproduction mediated by iNOS, thereby activating the cGAS/STING pathway, triggering inflammatory responses, and ultimately leading to coronary artery endothelial cell damage and myocardial dysfunction. iNOS is a key factor in the abnormal production of NO, which is the core driving force for mitochondrial damage and mtDNA release. The cGAS/STING pathway is mainly involved in the transmission of damage signals rather than the initiation process. Combined inhibition of iNOS and STING can synergistically improve mitochondrial function and inhibit inflammation, significantly reducing damage. This indicates that the NO-cGAS/STING pathway and inflammation are core mechanisms underlying hypoglycemia-induced vascular endothelial damage and are also potential therapeutic targets, providing a theoretical basis for the intervention of related cardiovascular complications. This study had several limitations that need to be acknowledged. First, direct molecular or biochemical evidence linking nitric oxide (NO) signaling to activation of the cGAS/STING pathway was not demonstrated in the present study. We did not confirm whether NO directly binds to cGAS/STING proteins, induces post‑translational modifications (such as S‑nitrosylation), or regulates cGAS enzymatic activity. Therefore, the causal relationship between NO and cGAS/STING activation remains to be further established. Second, the present results support the conclusion that NO indirectly regulates the cGAS/STING pathway by promoting mitochondrial damage and mtDNA release; however, other potential regulatory mechanisms cannot be excluded. Further in vitro biochemical experiments are required to verify the direct interaction and precise regulatory mechanism between NO and the cGAS/STING pathway. This will further confirm the causal relationship and enhance the completeness of the mechanistic investigation.

## Supporting information

S1 Data Raw experimental data supporting the findings of this study. (XLSX)
