# Supplementary material for: Role and mechanism of nitric oxide-regulated cGAS/STING pathway-mediated inflammatory response in hypoglycemia-induced coronary artery endothelial cell injury
Source: PLoS One. 2026 Jun 26;21(6):e0350983. doi: 10.1371/journal.pone.0350983 (PMC13308846; doi:10.1371/journal.pone.0350983)
Supplement: Attachment — Submitted filename: Response to Reviewers.docx [file pone.0350983.s003.docx]

PONE-D-25-31152

Role and Mechanism of Nitric Oxide-regulated cGAS/STING Pathway-mediated Inflammatory Response in Hypoglycemia-induced Coronary Artery Endothelial Cell Injury

PLOS ONE

Dear Dr. Luo,

Thank you for submitting your manuscript to PLOS ONE. After careful consideration, we feel that it has merit but does not fully meet PLOS ONE’s publication criteria as it currently stands. Therefore, we invite you to submit a revised version of the manuscript that addresses the points raised during the review process.

Please submit your revised manuscript by Sep 13 2025 11:59PM. If you will need more time than this to complete your revisions, please reply to this message or contact the journal office at [plosone@plos.org](mailto:plosone@plos.org). When you're ready to submit your revision, log on to https://www.editorialmanager.com/pone/ and select the 'Submissions Needing Revision' folder to locate your manuscript file.

Please include the following items when submitting your revised manuscript:

- A rebuttal letter that responds to each point raised by the academic editor and reviewer(s). You should upload this letter as a separate file labeled 'Response to Reviewers'.
- A marked-up copy of your manuscript that highlights changes made to the original version. You should upload this as a separate file labeled 'Revised Manuscript with Track Changes'.
- An unmarked version of your revised paper without tracked changes. You should upload this as a separate file labeled 'Manuscript'.

If you would like to make changes to your financial disclosure, please include your updated statement in your cover letter. Guidelines for resubmitting your figure files are available below the reviewer comments at the end of this letter.

If applicable, we recommend that you deposit your laboratory protocols in protocols.io to enhance the reproducibility of your results. Protocols.io assigns your protocol its own identifier (DOI) so that it can be cited independently in the future. For instructions see: <https://journals.plos.org/plosone/s/submission-guidelines#loc-laboratory-protocols>. Additionally, PLOS ONE offers an option for publishing peer-reviewed Lab Protocol articles, which describe protocols hosted on protocols.io. Read more information on sharing protocols at <https://plos.org/protocols?utm_medium=editorial-email&utm_source=authorletters&utm_campaign=protocols>.

We look forward to receiving your revised manuscript.

Kind regards,

Milad Khorasani, PhD
Reply: Thank you for informing us of the review results and suggestions for revision of the manuscript. We will prepare the revised manuscript as required and submit it through (https://www.editorialmanager.com/pone/) before 23:59 on September 13, 2025. We will also upload three files: "Response to Reviewers," "Revised Manuscript with Track Changes," and "Manuscript." We look forward to your review of the revised manuscript.

Academic Editor
PLOS ONE

Journal Requirements:

When submitting your revision, we need you to address these additional requirements.

1. Please ensure that your manuscript meets PLOS ONE's style requirements, including those for file naming. The PLOS ONE style templates can be found at

https://journals.plos.org/plosone/s/file?id=wjVg/PLOSOne_formatting_sample_main_body.pdf and

<https://journals.plos.org/plosone/s/file?id=ba62/PLOSOne_formatting_sample_title_authors_affiliations.pdf>

Reply: We have revised the manuscript in accordance with the formatting requirements of PLOS ONE, including file naming, and have referred to the provided style template.

2. To comply with PLOS One submissions requirements, in your Methods section, please provide additional information regarding the experiments involving animals and ensure you have included details on (1) methods of sacrifice, (2) methods of anesthesia and/or analgesia, and (3) efforts to alleviate suffering.

Reply: We have added the relevant information about the animal experiments, including the methods of sacrifice, anesthesia and/or analgesia, and measures to alleviate suffering, in the “Methods” section.

3. Thank you for stating the following financial disclosure: [This study is supported by the Science and Technology Program Projects in Social and Livelihood Areas in Bishan District of Chongqing Municipality（BSKJ2024063].

Please state what role the funders took in the study.  If the funders had no role, please state: ""The funders had no role in study design, data collection and analysis, decision to publish, or preparation of the manuscript.""

If this statement is not correct you must amend it as needed.

Please include this amended Role of Funder statement in your cover letter; we will change the online submission form on your behalf.

Reply: This study was funded by the Science and Technology Program for Social and People's Livelihood in Bishan District, Chongqing (BSKJ2024063). The funder had no role in the design of the study, the collection and analysis of data, the decision to publish, or the preparation of the manuscript.

4. We note that your Data Availability Statement is currently as follows: [All relevant data are within the manuscript and its Supporting Information files.]

Please confirm at this time whether or not your submission contains all raw data required to replicate the results of your study. Authors must share the “minimal data set” for their submission. PLOS defines the minimal data set to consist of the data required to replicate all study findings reported in the article, as well as related metadata and methods (https://journals.plos.org/plosone/s/data-availability#loc-minimal-data-set-definition).

For example, authors should submit the following data:

- The values behind the means, standard deviations and other measures reported;
- The values used to build graphs;
- The points extracted from images for analysis.

Authors do not need to submit their entire data set if only a portion of the data was used in the reported study.

If your submission does not contain these data, please either upload them as Supporting Information files or deposit them to a stable, public repository and provide us with the relevant URLs, DOIs, or accession numbers. For a list of recommended repositories, please see https://journals.plos.org/plosone/s/recommended-repositories.

If there are ethical or legal restrictions on sharing a de-identified data set, please explain them in detail (e.g., data contain potentially sensitive information, data are owned by a third-party organization, etc.) and who has imposed them (e.g., an ethics committee). Please also provide contact information for a data access committee, ethics committee, or other institutional body to which data requests may be sent. If data are owned by a third party, please indicate how others may request data access.

Reply: The de-identified dataset involved in this study can’t be made publicly available at present as it contains potentially sensitive information. Requests for access to the data can be made to the Animal Experiment Ethics Committee.

5. PLOS requires an ORCID iD for the corresponding author in Editorial Manager on papers submitted after December 6th, 2016. Please ensure that you have an ORCID iD and that it is validated in Editorial Manager. To do this, go to ‘Update my Information’ (in the upper left-hand corner of the main menu), and click on the Fetch/Validate link next to the ORCID field. This will take you to the ORCID site and allow you to create a new iD or authenticate a pre-existing iD in Editorial Manager.

Reply: The corresponding author has already obtained and verified the ORCID iD in Editorial Manager.

6. If the reviewer comments include a recommendation to cite specific previously published works, please review and evaluate these publications to determine whether they are relevant and should be cited. There is no requirement to cite these works unless the editor has indicated otherwise. 
Reply: We have evaluated the specific published works suggested in the review comments and decided whether to cite them based on their relevance.
[Note: HTML markup is below. Please do not edit.]

Reviewers' comments:

Reviewer's Responses to Questions

**Comments to the Author**

1. Is the manuscript technically sound, and do the data support the conclusions?

The manuscript must describe a technically sound piece of scientific research with data that supports the conclusions. Experiments must have been conducted rigorously, with appropriate controls, replication, and sample sizes. The conclusions must be drawn appropriately based on the data presented.

Reviewer #1: No

Reviewer #2: Yes

Reviewer #3: Partly

Reply: We have revised and improved the manuscript in response to Reviewer 1's concerns regarding the technical feasibility of the manuscript and the support of the data for the conclusions.

2. Has the statistical analysis been performed appropriately and rigorously?

Reviewer #1: I Don't Know

Reviewer #2: Yes

Reviewer #3: N/A

Reply: We have noted Reviewer 3's lack of comments on the statistical analysis and have ensured that there are no issues with the statistical analysis.

3. Have the authors made all data underlying the findings in their manuscript fully available?

The [PLOS Data policy](http://www.plosone.org/static/policies.action#sharing) requires authors to make all data underlying the findings described in their manuscript fully available without restriction, with rare exception (please refer to the Data Availability Statement in the manuscript PDF file). The data should be provided as part of the manuscript or its supporting information, or deposited to a public repository. For example, in addition to summary statistics, the data points behind means, medians and variance measures should be available. If there are restrictions on publicly sharing data—e.g. participant privacy or use of data from a third party—those must be specified.

Reviewer #1: No

Reviewer #2: Yes

Reviewer #3: No

Reply: We have supplemented and perfected the relevant data to ensure that all the basic data supporting the research findings have been fully provided.

4. Is the manuscript presented in an intelligible fashion and written in standard English?

PLOS ONE does not copyedit accepted manuscripts, so the language in submitted articles must be clear, correct, and unambiguous. Any typographical or grammatical errors should be corrected at revision, so please note any specific errors here.

Reviewer #1: Yes

Reviewer #2: Yes

Reviewer #3: Yes

Reply: Thank you to Reviewer 1 for acknowledging the clarity of expression in the manuscript.

5. Review Comments to the Author

Please use the space provided to explain your answers to the questions above. You may also include additional comments for the author, including concerns about dual publication, research ethics, or publication ethics. (Please upload your review as an attachment if it exceeds 20,000 characters)

Reviewer #1: This manuscript lack the standardized writing format. The results should present in Figure and show the detail data (for example OCR, Membrane potential, cGAS and STING expressions). Without the detail data, I am unable to evaluate the authenticity of the data in this paper.

Reply: The manuscript format has been standardized, and detailed data on OCR, membrane potential, and the expression of cGAS and STING have been supplemented and presented.

Reviewer #2: This research explains the content reasonably and completely. However, I have the following suggestions:
1. The important role of inflammatory cytokines including IFN-b, IL-6, and TNF-a in coronary artery endothelial cell injury should be discussed.
Reply: We have discussed the important roles of inflammatory cytokines such as IFN-β, IL-6, and TNF-α in coronary artery endothelial cell damage.
2. Please specify the axes of flow cytometric images in Figure 3.
Reply: The axes of the flow cytometry images in Figure 3 have been clearly labeled.
3. Statistical analysis used in this work should be explained in method section.

Reviewer #3: The article aims to elucidate the molecular mechanisms by which nitric oxide (NO) activates the cGAS/STING signaling pathway. To achieve this, the authors conducted extensive experimental studies using both in vitro approaches and animal models.
Reply: The statistical analysis methods used in this study have been described in the Methods section.
- In the "Experimental Animals"section, the author describes four experimental groups, including diabetic db/db mice, C57BL/6J mice, and iNOS-knockout and STING-knockout mice. Notably, C57BL/6J mice were used as the control group, despite also being described in the literature as models of diet-induced obesity and metabolic dysregulation. A brief justification for their selection as a control group should be provided in the Discussion section. Furthermore, a brief discussion on how the use of aged mice influences experimental outcomes would be beneficial
Reply: The rationale for choosing C57BL/6J mice as the control group has been explained in the Discussion section, and the impact of using aged mice on the experimental results has also been briefly discussed.
- It is also recommended to report the average body weight of the mice, given the obesity-prone nature of the models used.
Reply: The average body weight of the mice used has been supplemented.
- In the "Construction of the Diabetic Hypoglycemia Model"section, it remains unclear whether the hypoglycemic condition was induced in the knockout mouse groups. Clarification is needed.
Reply: It has been clearly stated whether the knockout mice group was induced into a hypoglycemic state.
- Figures 1 and 2 appear to present overlapping data. These should be consolidated into a single, comprehensive figure to improve clarity and reduce redundancy.
Reply: The overlapping data in Figures 1 and 2 have been combined into a single comprehensive figure to enhance clarity and reduce redundancy.
- In the "Primary Cell Isolation and Culture" section, the author should specify whether the coronary artery endothelial cells and cardiomyocytes were isolated following the induction of hypoglycemia. A timeline detailing each intervention in the animal model would enhance the clarity and reproducibility of the study.
Reply: It has been clearly stated whether the coronary artery endothelial cells and cardiomyocytes were isolated after hypoglycemia induction, and the timeline for each intervention in the animal model has been supplemented.
- The statistical methods employed in the study should be explicitly described, including tests used and significance thresholds.
Reply: The statistical methods used in this study, including the tests employed and the significance thresholds, have been clearly described.
- In the Results section, Table 1 refers to "Diabetic hypoglycemia," while Tables 2–5 use the term "Model group." Consistent nomenclature throughout the manuscript is recommended—preferably referencing specific mouse strains such as "db/db" or "C57BL/6J"—to avoid ambiguity.
Reply: The naming of tables in the Results section has been standardized, using specific mouse strain names such as “db/db” or “C57BL/6J” to avoid ambiguity.
- It is advisable to include representative images of mitochondrial structural analysis (microscopy) and gene expression analysis (e.g., western blot) to support the findings.
Reply: Images supporting the research findings have been added for mitochondrial structural analysis and gene expression analysis.
- In the cellular experiments, iNOS and STING inhibitors were used but not listed in the Materials and Methods section. These reagents should be clearly identified and described.
Reply: The iNOS and STING inhibitors used in the cell experiments have been clearly identified and described.
- In the Conclusions, the author asserts that "iNOS-mediated NO burst in endothelial cells under diabetic hypoglycemia induces mitochondrial injury and releases mtDNA." To substantiate this claim, the results from iNOS knockout mice presented in Tables 3 and 4 should be discussed, as they offer important insights into the contribution of NO to mitochondrial damage and mtDNA release.
Reply: The results of iNOS knockout mice in Tables 3 and 4 have been discussed in the Conclusions section to support the claim that "iNOS-mediated NO burst in endothelial cells under diabetic hypoglycemia induces mitochondrial injury and releases mtDNA".
- Finally, the manuscript does not address the expression levels of inflammatory mediators such as NF-κB, IL-1β, IL-8, ICAM-1, and GM-CSF. Including this data would provide a broader understanding of innate immune activation and inflammasome signaling in the experimental context.

Reply: The expression levels of inflammatory mediators such as NF-κB, IL-1β, IL-8, ICAM-1, and GM-CSF have been supplemented.

6. PLOS authors have the option to publish the peer review history of their article ([what does this mean?](https://journals.plos.org/plosone/s/editorial-and-peer-review-process#loc-peer-review-history)). If published, this will include your full peer review and any attached files.


If you choose “no”, your identity will remain anonymous but your review may still be made public.


**Do you want your identity to be public for this peer review?** For information about this choice, including consent withdrawal, please see our [Privacy Policy](https://www.plos.org/privacy-policy).

Reviewer #1: **Yes:** Fengqi Hu

Reviewer #2: No

Reviewer #3: No

Reply: We have taken note that Reviewer 1 has agreed to have their identity disclosed as Fengqi Hu.
