# Supplementary material for: Role and mechanism of nitric oxide-regulated cGAS/STING pathway-mediated inflammatory response in hypoglycemia-induced coronary artery endothelial cell injury
Source: PLoS One. 2026 Jun 26;21(6):e0350983. doi: 10.1371/journal.pone.0350983 (PMC13308846; doi:10.1371/journal.pone.0350983)
Supplement: Attachment — Submitted filename: Response_to_Reviewers_auresp_2.docx [file pone.0350983.s004.docx]

PONE-D-25-31152R1

Role and Mechanism of Nitric Oxide-regulated cGAS/STING Pathway-mediated Inflammatory Response in Hypoglycemia-induced Coronary Artery Endothelial Cell Injury

PLOS ONE

Dear Dr. Luo, 

Thank you for submitting your manuscript to PLOS ONE. After careful consideration, we feel that it has merit but does not fully meet PLOS ONE’s publication criteria as it currently stands. Therefore, we invite you to submit a revised version of the manuscript that addresses the points raised during the review process.

Please submit your revised manuscript by Dec 13 2025 11:59PM. If you will need more time than this to complete your revisions, please reply to this message or contact the journal office at [plosone@plos.org](mailto:plosone@plos.org). When you're ready to submit your revision, log on to https://www.editorialmanager.com/pone/ and select the 'Submissions Needing Revision' folder to locate your manuscript file.
Reply: Thank you for your feedback and guidance. We have completed the revision of the manuscript and will strictly adhere to the requirements to submit the revised version via the designated platform by 23:59 on December 13, 2025.

Please include the following items when submitting your revised manuscript:

- A rebuttal letter that responds to each point raised by the academic editor and reviewer(s). You should upload this letter as a separate file labeled 'Response to Reviewers'.
- A marked-up copy of your manuscript that highlights changes made to the original version. You should upload this as a separate file labeled 'Revised Manuscript with Track Changes'.
- An unmarked version of your revised paper without tracked changes. You should upload this as a separate file labeled 'Manuscript'.

If you would like to make changes to your financial disclosure, please include your updated statement in your cover letter. Guidelines for resubmitting your figure files are available below the reviewer comments at the end of this letter.
Reply: Thank you for your clear instructions. We have prepared the following documents as required, which will be submitted together with the revised manuscript: a rebuttal letter labeled "Response to Reviewers," addressing all comments from the academic editor and reviewers point by point; a revised manuscript with track changes labeled "Revised Manuscript with Track Changes," clearly indicating all modifications; and a clean version of the revised manuscript labeled "Manuscript." The financial disclosure information for this study remains unchanged, and relevant figure files will be submitted in accordance with subsequent guidelines.

If applicable, we recommend that you deposit your laboratory protocols in protocols.io to enhance the reproducibility of your results. Protocols.io assigns your protocol its own identifier (DOI) so that it can be cited independently in the future. For instructions see: <https://journals.plos.org/plosone/s/submission-guidelines#loc-laboratory-protocols>. Additionally, PLOS ONE offers an option for publishing peer-reviewed Lab Protocol articles, which describe protocols hosted on protocols.io. Read more information on sharing protocols at <https://plos.org/protocols?utm_medium=editorial-email&utm_source=authorletters&utm_campaign=protocols>.
Reply: Thank you for your valuable suggestion. Considering that some of the experimental protocols in this study involve internal laboratory technical details and relevant ethical requirements, they cannot be uploaded to the protocols.io platform at this time. We have provided a detailed description of the core experimental procedures, reagent specifications, operational parameters, and key steps in the manuscript to ensure the reproducibility of the research findings. Should any peers require further details regarding the experiments, we will provide necessary support in compliance with relevant regulations. We apologize for any inconvenience this may cause.

We look forward to receiving your revised manuscript.

Kind regards,

Milad Khorasani, PhD
Academic Editor
PLOS ONE

Journal Requirements:

If the reviewer comments include a recommendation to cite specific previously published works, please review and evaluate these publications to determine whether they are relevant and should be cited. There is no requirement to cite these works unless the editor has indicated otherwise. 
Reply: Thank you for your reminder and attention. We have carefully reviewed the reviewers' comments and confirmed that there are no specific recommendations to cite particular published literature. In the subsequent process, we will strictly adhere to the journal's requirements, citing only relevant and necessary literature in a standardized manner to ensure the rigor and relevance of the manuscript's references. The revised manuscript will be submitted as scheduled, and we thank you again for your guidance.

[Note: HTML markup is below. Please do not edit.]

Reviewers' comments:

Reviewer's Responses to Questions

**Comments to the Author**

1. If the authors have adequately addressed your comments raised in a previous round of review and you feel that this manuscript is now acceptable for publication, you may indicate that here to bypass the “Comments to the Author” section, enter your conflict of interest statement in the “Confidential to Editor” section, and submit your "Accept" recommendation.

Reviewer #4: All comments have been addressed

Reviewer #5: (No Response)

Reply: Thank you for confirming that we have adequately addressed all previous comments. We have refined the manuscript as required and will submit the revised version on schedule.

2. Is the manuscript technically sound, and do the data support the conclusions?

The manuscript must describe a technically sound piece of scientific research with data that supports the conclusions. Experiments must have been conducted rigorously, with appropriate controls, replication, and sample sizes. The conclusions must be drawn appropriately based on the data presented.

Reviewer #4: (No Response)

Reviewer #5: Yes

Reply: We appreciate your acknowledgment of the rationality of the technical approach, the rigor of the experimental design, and the relevance of the data to the conclusions in this study. We will maintain the current scientific framework for the final submission.

3. Has the statistical analysis been performed appropriately and rigorously?

Reviewer #4: (No Response)

Reviewer #5: Yes

Reply: We thank you for your positive assessment of the selection and rigorous implementation of statistical methods in this study. The relevant statistical details have been presented in the manuscript in accordance with standard practices.

4. Have the authors made all data underlying the findings in their manuscript fully available?

The [PLOS Data policy](http://www.plosone.org/static/policies.action#sharing) requires authors to make all data underlying the findings described in their manuscript fully available without restriction, with rare exception (please refer to the Data Availability Statement in the manuscript PDF file). The data should be provided as part of the manuscript or its supporting information, or deposited to a public repository. For example, in addition to summary statistics, the data points behind means, medians and variance measures should be available. If there are restrictions on publicly sharing data—e.g. participant privacy or use of data from a third party—those must be specified.

Reviewer #4: (No Response)

Reviewer #5: Yes

Reply: We appreciate your recognition of the data accessibility in this study. The relevant data have been disclosed in compliance with the PLOS Data Policy, ensuring adherence to the journal's requirements.

5. Is the manuscript presented in an intelligible fashion and written in standard English?

PLOS ONE does not copyedit accepted manuscripts, so the language in submitted articles must be clear, correct, and unambiguous. Any typographical or grammatical errors should be corrected at revision, so please note any specific errors here.

Reviewer #4: (No Response)

Reviewer #5: Yes

Reply: We thank you for your affirmation regarding the clarity of the manuscript and the standardization of the language. We have reviewed and corrected any potential formatting or grammatical issues to ensure the manuscript is comprehensible and accurate.

6. Review Comments to the Author

Please use the space provided to explain your answers to the questions above. You may also include additional comments for the author, including concerns about dual publication, research ethics, or publication ethics. (Please upload your review as an attachment if it exceeds 20,000 characters)

Reviewer #4: As a newly invited reviewer for the revised version of this manuscript, I have carefully evaluated the authors’ responses to the previous reviewers’ comments as well as the revised manuscript itself. t is evident that the authors have addressed the prior concerns comprehensively and improved the clarity, methodology, and overall presentation of the paper. The scientific content appears sound, and the revisions adequately resolve the major issues raised during the initial review. Therefore, in my opinion, the manuscript now meets the publication standards of PLOS ONE and can be accepted in its current form.

Reply: We appreciate your positive feedback. We have fully addressed all previous review comments, enhanced the manuscript's clarity, methodological descriptions, and overall presentation, and will submit the final revised version as required by the deadline.

Reviewer #5: Thanks to the authors for revising the manuscript. Revising the following can help improve the manuscript.
#1 Please moderate causal statements such as “NO activates the cGAS/STING pathway” to “NO may contribute to the activation…” since direct biochemical evidence (e.g., nitrosylation or co-localization assays) was not provided. Add one short sentence acknowledging this limitation in the Discussion.

Reply: Absolute causal statements such as "NO activates the cGAS/STING pathway" have been revised to more speculative phrasing like "NO may contribute to the activation of the cGAS/STING pathway." Additional sentences have been added to the Discussion section to address the associated limitations.
#2 Clarify abbreviations at first appearance. Ensure that all abbreviations (e.g., OCR, mPTP, RCR) are defined upon first mention in both the main text and figure legends.

Reply: The entire text has been thoroughly checked. All abbreviations (including OCR, mPTP, RCR) are now defined upon their first occurrence in both the main text and figure legends to ensure standardized presentation.

7. PLOS authors have the option to publish the peer review history of their article ([what does this mean?](https://journals.plos.org/plosone/s/editorial-and-peer-review-process#loc-peer-review-history)). If published, this will include your full peer review and any attached files.


If you choose “no”, your identity will remain anonymous but your review may still be made public.


**Do you want your identity to be public for this peer review?** For information about this choice, including consent withdrawal, please see our [Privacy Policy](https://www.plos.org/privacy-policy).

Reviewer #4: No

Reviewer #5: No

Reply: Thank you for informing us of the relevant policy. We respect your decision to publish the review comments anonymously.
