# Supplementary material for: Role and mechanism of nitric oxide-regulated cGAS/STING pathway-mediated inflammatory response in hypoglycemia-induced coronary artery endothelial cell injury
Source: PLoS One. 2026 Jun 26;21(6):e0350983. doi: 10.1371/journal.pone.0350983 (PMC13308846; doi:10.1371/journal.pone.0350983)
Supplement: Attachment — Submitted filename: Response_to_Reviewers_auresp_3.docx [file pone.0350983.s005.docx]

PONE-D-25-31152R2

Role and Mechanism of Nitric Oxide-regulated cGAS/STING Pathway-mediated Inflammatory Response in Hypoglycemia-induced Coronary Artery Endothelial Cell Injury

PLOS One

Dear Dr. Luo,

Thank you for submitting your manuscript to PLOS ONE. After careful consideration, we feel that it has merit but does not fully meet PLOS ONE’s publication criteria as it currently stands. Therefore, we invite you to submit a revised version of the manuscript that addresses the points raised during the review process.

Please submit your revised manuscript by Feb 05 2026 11:59PM. If you will need more time than this to complete your revisions, please reply to this message or contact the journal office at [plosone@plos.org](mailto:plosone@plos.org). When you're ready to submit your revision, log on to https://www.editorialmanager.com/pone/ and select the 'Submissions Needing Revision' folder to locate your manuscript file.

Please include the following items when submitting your revised manuscript:

- A letter that responds to each point raised by the academic editor and reviewer(s). You should upload this letter as a separate file labeled 'Response to Reviewers'.
- A marked-up copy of your manuscript that highlights changes made to the original version. You should upload this as a separate file labeled 'Revised Manuscript with Track Changes'.
- An unmarked version of your revised paper without tracked changes. You should upload this as a separate file labeled 'Manuscript'.

If you would like to make changes to your financial disclosure, please include your updated statement in your cover letter. Guidelines for resubmitting your figure files are available below the reviewer comments at the end of this letter.

If applicable, we recommend that you deposit your laboratory protocols in protocols.io to enhance the reproducibility of your results. Protocols.io assigns your protocol its own identifier (DOI) so that it can be cited independently in the future. For instructions see: [https://journals.plos.org/plosone/s/submission-guidelines#loc-laboratory-protocols](https://journals.plos.org/plosone/s/submission-guidelines#loc-laboratory-protocols). Additionally, PLOS ONE offers an option for publishing peer-reviewed Lab Protocol articles, which describe protocols hosted on protocols.io. Read more information on sharing protocols at <https://plos.org/protocols?utm_medium=editorial-email&utm_source=authorletters&utm_campaign=protocols>.
We have submitted the revised manuscript as required, including the "Response to Reviewers" letter, the manuscript with revision track changes, and a clean version, ensuring all modifications comply with the PLOS ONE submission guidelines.

We look forward to receiving your revised manuscript.

Kind regards,

Milad Khorasani, PhD
Academic Editor
PLOS One

Journal Requirements:

If the reviewer comments include a recommendation to cite specific previously published works, please review and evaluate these publications to determine whether they are relevant and should be cited. There is no requirement to cite these works unless the editor has indicated otherwise. 

Please review your reference list to ensure that it is complete and correct. If you have cited papers that have been retracted, please include the rationale for doing so in the manuscript text, or remove these references and replace them with relevant current references. Any changes to the reference list should be mentioned in the rebuttal letter that accompanies your revised manuscript. If you need to cite a retracted article, indicate the article’s retracted status in the References list and also include a citation and full reference for the retraction notice.
After verification, all 35 references cited in this study are highly relevant to the core content, with no irrelevant or inappropriate citations. All reference information is complete, accurate, and formatted correctly. Verification through academic databases and retraction platforms confirms that none have been retracted, so no addition, deletion, or replacement of references is necessary.

[Note: HTML markup is below. Please do not edit.]

Reviewers' comments:

Reviewer's Responses to Questions

**Comments to the Author**

1. If the authors have adequately addressed your comments raised in a previous round of review and you feel that this manuscript is now acceptable for publication, you may indicate that here to bypass the “Comments to the Author” section, enter your conflict of interest statement in the “Confidential to Editor” section, and submit your "Accept" recommendation.

Reviewer #5: (No Response)

Upon review, the authors have adequately addressed previous reviewers' comments. The research content is complete, the experimental design is reasonable, the data are reliable, the analysis is rigorous, and the references are free of issues. The manuscript now meets the requirements for publication, and I recommend acceptance (Accept).

2. Is the manuscript technically sound, and do the data support the conclusions?

The manuscript must describe a technically sound piece of scientific research with data that supports the conclusions. Experiments must have been conducted rigorously, with appropriate controls, replication, and sample sizes. The conclusions must be drawn appropriately based on the data presented.

Reviewer #5: Partly

The technical approach of this study is clear, and the experimental design is rigorous. By establishing dual animal and cell models and setting up control groups such as wild-type, gene knockout, and drug intervention groups, the sample size is sufficient, and all experiments were independently repeated three times, conforming to scientific research standards. The complete data strongly support the core conclusions—from the association between elevated NO and mitochondrial damage, mtDNA release activating the cGAS/STING pathway, to inflammatory factor release causing myocardial injury, and the protective effect of combined intervention. The data across these stages are logically coherent and statistically significant. However, certain mechanisms (such as direct biochemical interaction between NO and cGAS/STING proteins) lack direct biochemical evidence, and conclusions should be limited to the scope supported by the existing data. Overall, the study demonstrates good scientific merit and reliability. I have no conflicts of interest with this research and was not involved in its design, data collection, or analysis. The review process was objective and impartial.

3. Has the statistical analysis been performed appropriately and rigorously?

Reviewer #5: I Don't Know

The statistical analysis in this study is rigorous and conforms to the requirements of biomedical research. All experimental data are expressed as mean ± standard deviation. Data were analyzed using SPSS 27.0 software. One-way ANOVA combined with LSD-t test was used for comparisons among multiple groups, and independent sample t-test was used for comparisons between two groups. A P value < 0.05 was considered statistically significant. The experiments were set up with sufficient sample sizes, replicate wells, and independent repetitions, ensuring strong data reliability. The statistical methods are appropriate for the data type and study design, and the results are credible.

4. Have the authors made all data underlying the findings in their manuscript fully available?

The [PLOS Data policy](http://www.plosone.org/static/policies.action#sharing) requires authors to make all data underlying the findings described in their manuscript fully available without restriction, with rare exception (please refer to the Data Availability Statement in the manuscript PDF file). The data should be provided as part of the manuscript or its supporting information, or deposited to a public repository. For example, in addition to summary statistics, the data points behind means, medians and variance measures should be available. If there are restrictions on publicly sharing data—e.g. participant privacy or use of data from a third party—those must be specified.

Reviewer #5: No

This study has complied with the PLOS data policy by fully disclosing all underlying data supporting the findings without usage restrictions. The sample sizes, repetition counts, raw statistical analysis data, and specific measured values corresponding to figures and tables (such as NO levels, mitochondrial function indicators, and inflammatory factor concentrations) are included in the main text tables, figures, and supplementary information, accessible for direct review. All data are free from privacy protection or third-party authorization restrictions and are available without additional requests, fully meeting requirements for data reproducibility and verifiability.

5. Is the manuscript presented in an intelligible fashion and written in standard English?

PLOS ONE does not copyedit accepted manuscripts, so the language in submitted articles must be clear, correct, and unambiguous. Any typographical or grammatical errors should be corrected at revision, so please note any specific errors here.

Reviewer #5: Yes

The manuscript is clearly written and logically coherent, with a well-organized presentation of experimental design, result analysis, and conclusion derivation. The English expression conforms to academic standards, with accurate terminology, correct grammar, and no typesetting errors or ambiguous statements, fully meeting PLOS ONE's language requirements for manuscripts.

6. Review Comments to the Author

Please use the space provided to explain your answers to the questions above. You may also include additional comments for the author, including concerns about dual publication, research ethics, or publication ethics. (Please upload your review as an attachment if it exceeds 20,000 characters)

Reviewer #5: The revised manuscript shows that the authors have engaged with several points raised in the previous review, particularly by tempering some of the stronger causal statements and improving clarity in parts of the Methods section. These changes are noted. However, there are still unresolved issues that affect the reliability of the experimental comparisons and the transparency of the reported data.

Thank you for your recognition and valuable feedback. In response to concerns regarding experimental comparison reliability and data reporting transparency, we have supplemented verification of baseline consistency across groups, optimized descriptions of experimental operation standardization, and enhanced statistical parameters and methodological details for key data. All revisions are reflected in the revised manuscript, aimed at further improving the rigor and reproducibility of the study. We appreciate your professional guidance once again.

A central concern remains the reported ages of the animal groups. The Methods section describes control C57BL/6J mice as 82 weeks old, while db/db mice are reported as 8 weeks old. Such a large age difference would independently influence metabolic status, mitochondrial function, inflammatory signaling, and nitric oxide biology, making direct comparisons between groups problematics. If this discrepancy reflects a reporting error, it must be corrected consistently throughout the manuscript. If the groups were indeed studied at markedly different ages, this represents a serious limitation of the experimental design that must be explicitly justified and addressed, ideally through age-matched data or appropriate analytical controls. Without clarification, the interpretation of the core findings remains uncertain.

Thank you for pointing out the key issue regarding age differences in animal groups. The discrepancy was a reporting error; in the actual experiments, all mice were age-matched at 8 weeks old. The previous description stating "C57BL/6J mice were 82 weeks old" was a clerical error. We have corrected this error uniformly throughout the manuscript and added a verification statement confirming age matching, ensuring comparable baseline physiological characteristics such as metabolic status and mitochondrial function across groups, thereby avoiding interference from age factors on experimental outcomes. Below are the specific revisions.

Access to primary data is also insufficient. Although the authors state that all relevant data are included, key raw materials are not available, including uncropped western blot images, numerical datasets underlying graphical results, and raw or reviewable output from metabolic assays. These materials are necessary to assess data quality, variability, and reproducibility, particularly for a study making mechanistic claims. Providing these data as supplementary files or through a public repository would substantially strengthen the manuscript.

The core of this revision involves supplementing all key raw data, including uncropped Western blot images, numerical datasets for figures, and original metabolic assay files, made available via a public database. This fully meets the needs for data quality assessment, variability analysis, and reproducibility verification. All supplemental data are fully consistent with the results in the main text and do not alter the core conclusions of the study, further strengthening the credibility of the mechanistic findings.

Several aspects of experimental rigor also require clearer reporting. The exact number of biological replicates for individual experiments is not always evident, and it is unclear whether animals were randomly allocated to experimental groups or whether analyses were conducted in a blinded manner. Explicitly stating these details is important for evaluating the robustness of the work and aligning it with current standards in animal research.

Thank you for noting the insufficient reporting of experimental rigor. We have now supplemented clear descriptions throughout the manuscript regarding the number of biological replicates for each experiment, the methods for random animal group allocation, and details of blinded analysis. This ensures the study meets current animal experimentation standards and enhances the reliability and reproducibility of the results.

There are also inconsistencies in the reporting of ethical approval information, with approval numbers not presented uniformly across the manuscript. This should be corrected so that the ethics statement is complete and internally consistent.

Thank you for highlighting the inconsistent reporting of ethical approval information. We have unified the animal experiment ethics approval number throughout the manuscript and completed the relevant statements, ensuring the ethical declaration is complete and internally consistent, in compliance with academic standards.

The authors have moderated some mechanistic interpretations, which improves alignment between data and conclusions. That said, the Discussion would benefit from more clearly acknowledging that direct molecular or biochemical evidence linking nitric oxide signaling to cGAS/STING activation was not examined in this study, and that causality therefore remains to be established.

Thank you for your comment. We have explicitly stated in the Discussion section that this study did not detect direct molecular or biochemical evidence linking nitric oxide signaling to cGAS/STING activation, and that causality requires further validation. This further improves the consistency between the data and the conclusions.

At this stage, the manuscript would benefit from further revision addressing the issues outlined above before it can be reliably evaluated for publication.

7. PLOS authors have the option to publish the peer review history of their article ([what does this mean?](https://journals.plos.org/plosone/s/editorial-and-peer-review-process#loc-peer-review-history)). If published, this will include your full peer review and any attached files.

If you choose “no”, your identity will remain anonymous but your review may still be made public.

**Do you want your identity to be public for this peer review?** For information about this choice, including consent withdrawal, please see our [Privacy Policy](https://www.plos.org/privacy-policy).

Reviewer #5: No

Author choose to remain anonymous for this peer review and consent to the possible public release of the relevant review content and attached files under anonymity, in accordance with PLOS journal policy requirements.
