# Supplementary material for: Role and mechanism of nitric oxide-regulated cGAS/STING pathway-mediated inflammatory response in hypoglycemia-induced coronary artery endothelial cell injury
Source: PLoS One. 2026 Jun 26;21(6):e0350983. doi: 10.1371/journal.pone.0350983 (PMC13308846; doi:10.1371/journal.pone.0350983)
Supplement: Attachment — Submitted filename: Response to Reviewers.doc [file pone.0350983.s006.doc]

**Response to Reviewers**

We greatly appreciate the academic editor and reviewers for their constructive comments and suggestions, which have significantly helped us improve the quality of our manuscript. We have carefully addressed all comments and revised the manuscript accordingly. Below is our point-by-point response.

1. Age discrepancy between control (82 weeks) and db/db (8 weeks) mice

Reply: We apologize for the typographical error. The age “82 weeks old” has been corrected to 8±2 weeks old, consistent with all other experimental groups.

2. Provision of key raw data

Reply: We have provided uncropped original western blot images for cGAS and STING, as well as complete numerical datasets in the supplementary materials to ensure transparency and reproducibility.

3. Standardization of ethical approval information

Reply: We have standardized the ethical approval statement into a consistent formal format throughout the manuscript.

4. Acknowledgment of lacking direct molecular evidence

Reply: We have revised the discussion and conclusion sections to clearly state that direct molecular or biochemical evidence linking NO signaling to cGAS/STING activation was not demonstrated, and that causality remains to be established.
