# Supplementary material for: Role and mechanism of nitric oxide-regulated cGAS/STING pathway-mediated inflammatory response in hypoglycemia-induced coronary artery endothelial cell injury
Source: PLoS One. 2026 Jun 26;21(6):e0350983. doi: 10.1371/journal.pone.0350983 (PMC13308846; doi:10.1371/journal.pone.0350983)
Supplement: Peer review history

# Peer review: Decision Letter 0

**DOI:** 10.1371/journal.pone.0350983.r001

**Authors:** Milad Khorasani

30 Jul 2025

PONE-D-25-31152

Role and Mechanism of Nitric Oxide-regulated cGAS/STING Pathway-mediated Inflammatory Response in Hypoglycemia-induced Coronary Artery Endothelial Cell Injury

PLOS ONE

Dear Dr. Luo,

Thank you for submitting your manuscript to PLOS ONE. After careful consideration, we feel that it has merit but does not fully meet PLOS ONE’s publication criteria as it currently stands. Therefore, we invite you to submit a revised version of the manuscript that addresses the points raised during the review process.

Please submit your revised manuscript by Sep 13 2025 11:59PM. If you will need more time than this to complete your revisions, please reply to this message or contact the journal office at plosone@plos.org. When you're ready to submit your revision, log on to https://www.editorialmanager.com/pone/ and select the 'Submissions Needing Revision' folder to locate your manuscript file.

Please include the following items when submitting your revised manuscript:

- A rebuttal letter that responds to each point raised by the academic editor and reviewer(s). You should upload this letter as a separate file labeled 'Response to Reviewers'.
- A marked-up copy of your manuscript that highlights changes made to the original version. You should upload this as a separate file labeled 'Revised Manuscript with Track Changes'.
- An unmarked version of your revised paper without tracked changes. You should upload this as a separate file labeled 'Manuscript'.

If you would like to make changes to your financial disclosure, please include your updated statement in your cover letter. Guidelines for resubmitting your figure files are available below the reviewer comments at the end of this letter.

If applicable, we recommend that you deposit your laboratory protocols in protocols.io to enhance the reproducibility of your results. Protocols.io assigns your protocol its own identifier (DOI) so that it can be cited independently in the future. For instructions see: https://journals.plos.org/plosone/s/submission-guidelines#loc-laboratory-protocols. Additionally, PLOS ONE offers an option for publishing peer-reviewed Lab Protocol articles, which describe protocols hosted on protocols.io. Read more information on sharing protocols at https://plos.org/protocols?utm_medium=editorial-email&utm_source=authorletters&utm_campaign=protocols.

We look forward to receiving your revised manuscript.

Kind regards,

Milad Khorasani, PhD

Academic Editor

PLOS ONE

Journal Requirements:

When submitting your revision, we need you to address these additional requirements.

1. Please ensure that your manuscript meets PLOS ONE's style requirements, including those for file naming. The PLOS ONE style templates can be found at

https://journals.plos.org/plosone/s/file?id=wjVg/PLOSOne_formatting_sample_main_body.pdf and

https://journals.plos.org/plosone/s/file?id=ba62/PLOSOne_formatting_sample_title_authors_affiliations.pdf

2. To comply with PLOS One submissions requirements, in your Methods section, please provide additional information regarding the experiments involving animals and ensure you have included details on (1) methods of sacrifice, (2) methods of anesthesia and/or analgesia, and (3) efforts to alleviate suffering.

3. Thank you for stating the following financial disclosure: [This study is supported by the Science and Technology Program Projects in Social and Livelihood Areas in Bishan District of Chongqing Municipality（BSKJ2024063].

Please state what role the funders took in the study.  If the funders had no role, please state: ""The funders had no role in study design, data collection and analysis, decision to publish, or preparation of the manuscript.""

If this statement is not correct you must amend it as needed.

Please include this amended Role of Funder statement in your cover letter; we will change the online submission form on your behalf.

4. We note that your Data Availability Statement is currently as follows: [All relevant data are within the manuscript and its Supporting Information files.]

Please confirm at this time whether or not your submission contains all raw data required to replicate the results of your study. Authors must share the “minimal data set” for their submission. PLOS defines the minimal data set to consist of the data required to replicate all study findings reported in the article, as well as related metadata and methods (https://journals.plos.org/plosone/s/data-availability#loc-minimal-data-set-definition).

For example, authors should submit the following data:

- The values behind the means, standard deviations and other measures reported;

- The values used to build graphs;

- The points extracted from images for analysis.

Authors do not need to submit their entire data set if only a portion of the data was used in the reported study.

If your submission does not contain these data, please either upload them as Supporting Information files or deposit them to a stable, public repository and provide us with the relevant URLs, DOIs, or accession numbers. For a list of recommended repositories, please see https://journals.plos.org/plosone/s/recommended-repositories.

If there are ethical or legal restrictions on sharing a de-identified data set, please explain them in detail (e.g., data contain potentially sensitive information, data are owned by a third-party organization, etc.) and who has imposed them (e.g., an ethics committee). Please also provide contact information for a data access committee, ethics committee, or other institutional body to which data requests may be sent. If data are owned by a third party, please indicate how others may request data access.

5. PLOS requires an ORCID iD for the corresponding author in Editorial Manager on papers submitted after December 6th, 2016. Please ensure that you have an ORCID iD and that it is validated in Editorial Manager. To do this, go to ‘Update my Information’ (in the upper left-hand corner of the main menu), and click on the Fetch/Validate link next to the ORCID field. This will take you to the ORCID site and allow you to create a new iD or authenticate a pre-existing iD in Editorial Manager.

6. If the reviewer comments include a recommendation to cite specific previously published works, please review and evaluate these publications to determine whether they are relevant and should be cited. There is no requirement to cite these works unless the editor has indicated otherwise.

[Note: HTML markup is below. Please do not edit.]

Reviewers' comments:

Reviewer's Responses to Questions

**Comments to the Author**

1. Is the manuscript technically sound, and do the data support the conclusions?

The manuscript must describe a technically sound piece of scientific research with data that supports the conclusions. Experiments must have been conducted rigorously, with appropriate controls, replication, and sample sizes. The conclusions must be drawn appropriately based on the data presented.

Reviewer #1: No

Reviewer #2: Yes

Reviewer #3: Partly

**********

2. Has the statistical analysis been performed appropriately and rigorously?

Reviewer #1: I Don't Know

Reviewer #2: Yes

Reviewer #3: N/A

**********

3. Have the authors made all data underlying the findings in their manuscript fully available?

The PLOS Data policy requires authors to make all data underlying the findings described in their manuscript fully available without restriction, with rare exception (please refer to the Data Availability Statement in the manuscript PDF file). The data should be provided as part of the manuscript or its supporting information, or deposited to a public repository. For example, in addition to summary statistics, the data points behind means, medians and variance measures should be available. If there are restrictions on publicly sharing data—e.g. participant privacy or use of data from a third party—those must be specified.

Reviewer #1: No

Reviewer #2: Yes

Reviewer #3: No

**********

4. Is the manuscript presented in an intelligible fashion and written in standard English?

PLOS ONE does not copyedit accepted manuscripts, so the language in submitted articles must be clear, correct, and unambiguous. Any typographical or grammatical errors should be corrected at revision, so please note any specific errors here.

Reviewer #1: Yes

Reviewer #2: Yes

Reviewer #3: Yes

**********

5. Review Comments to the Author

Please use the space provided to explain your answers to the questions above. You may also include additional comments for the author, including concerns about dual publication, research ethics, or publication ethics. (Please upload your review as an attachment if it exceeds 20,000 characters)

Reviewer #1: This manuscript lack the standardized writing format. The results should present in Figure and show the detail data (for example OCR, Membrane potential, cGAS and STING expressions). Without the detail data, I am unable to evaluate the authenticity of the data in this paper.

Reviewer #2: This research explains the content reasonably and completely. However, I have the following suggestions:

1. The important role of inflammatory cytokines including IFN-b, IL-6, and TNF-a in coronary artery endothelial cell injury should be discussed.

2. Please specify the axes of flow cytometric images in Figure 3.

3. Statistical analysis used in this work should be explained in method section.

Reviewer #3: The article aims to elucidate the molecular mechanisms by which nitric oxide (NO) activates the cGAS/STING signaling pathway. To achieve this, the authors conducted extensive experimental studies using both in vitro approaches and animal models.

- In the "Experimental Animals"section, the author describes four experimental groups, including diabetic db/db mice, C57BL/6J mice, and iNOS-knockout and STING-knockout mice. Notably, C57BL/6J mice were used as the control group, despite also being described in the literature as models of diet-induced obesity and metabolic dysregulation. A brief justification for their selection as a control group should be provided in the Discussion section. Furthermore, a brief discussion on how the use of aged mice influences experimental outcomes would be beneficial

- It is also recommended to report the average body weight of the mice, given the obesity-prone nature of the models used.

- In the "Construction of the Diabetic Hypoglycemia Model"section, it remains unclear whether the hypoglycemic condition was induced in the knockout mouse groups. Clarification is needed.

- Figures 1 and 2 appear to present overlapping data. These should be consolidated into a single, comprehensive figure to improve clarity and reduce redundancy.

- In the "Primary Cell Isolation and Culture" section, the author should specify whether the coronary artery endothelial cells and cardiomyocytes were isolated following the induction of hypoglycemia. A timeline detailing each intervention in the animal model would enhance the clarity and reproducibility of the study.

- The statistical methods employed in the study should be explicitly described, including tests used and significance thresholds.

- In the Results section, Table 1 refers to "Diabetic hypoglycemia," while Tables 2–5 use the term "Model group." Consistent nomenclature throughout the manuscript is recommended—preferably referencing specific mouse strains such as "db/db" or "C57BL/6J"—to avoid ambiguity.

- It is advisable to include representative images of mitochondrial structural analysis (microscopy) and gene expression analysis (e.g., western blot) to support the findings.

- In the cellular experiments, iNOS and STING inhibitors were used but not listed in the Materials and Methods section. These reagents should be clearly identified and described.

- In the Conclusions, the author asserts that "iNOS-mediated NO burst in endothelial cells under diabetic hypoglycemia induces mitochondrial injury and releases mtDNA." To substantiate this claim, the results from iNOS knockout mice presented in Tables 3 and 4 should be discussed, as they offer important insights into the contribution of NO to mitochondrial damage and mtDNA release.

- Finally, the manuscript does not address the expression levels of inflammatory mediators such as NF-κB, IL-1β, IL-8, ICAM-1, and GM-CSF. Including this data would provide a broader understanding of innate immune activation and inflammasome signaling in the experimental context.

**********

6. PLOS authors have the option to publish the peer review history of their article (what does this mean?). If published, this will include your full peer review and any attached files.

If you choose “no”, your identity will remain anonymous but your review may still be made public.

**Do you want your identity to be public for this peer review?**  For information about this choice, including consent withdrawal, please see our Privacy Policy.

Reviewer #1: **Yes:**  Fengqi Hu

Reviewer #2: No

Reviewer #3: No

**********

[NOTE: If reviewer comments were submitted as an attachment file, they will be attached to this email and accessible via the submission site. Please log into your account, locate the manuscript record, and check for the action link "View Attachments". If this link does not appear, there are no attachment files.]

While revising your submission, please upload your figure files to the Preflight Analysis and Conversion Engine (PACE) digital diagnostic tool, https://pacev2.apexcovantage.com/. PACE helps ensure that figures meet PLOS requirements. To use PACE, you must first register as a user. Registration is free. Then, login and navigate to the UPLOAD tab, where you will find detailed instructions on how to use the tool. If you encounter any issues or have any questions when using PACE, please email PLOS at figures@plos.org. Please note that Supporting Information files do not need this step.

# Author comment: Author response to Decision Letter 1

**DOI:** 10.1371/journal.pone.0350983.r002

9 Sep 2025

We look forward to receiving your revised manuscript.

Kind regards,

Milad Khorasani, PhD

Reply: Thank you for informing us of the review results and suggestions for revision of the manuscript. We will prepare the revised manuscript as required and submit it through (https://www.editorialmanager.com/pone/) before 23:59 on September 13, 2025. We will also upload three files: "Response to Reviewers," "Revised Manuscript with Track Changes," and "Manuscript." We look forward to your review of the revised manuscript.

Academic Editor

PLOS ONE

Journal Requirements:

When submitting your revision, we need you to address these additional requirements.

1. Please ensure that your manuscript meets PLOS ONE's style requirements, including those for file naming. The PLOS ONE style templates can be found at

https://journals.plos.org/plosone/s/file?id=wjVg/PLOSOne_formatting_sample_main_body.pdf and

https://journals.plos.org/plosone/s/file?id=ba62/PLOSOne_formatting_sample_title_authors_affiliations.pdf

Reply: We have revised the manuscript in accordance with the formatting requirements of PLOS ONE, including file naming, and have referred to the provided style template.

2. To comply with PLOS One submissions requirements, in your Methods section, please provide additional information regarding the experiments involving animals and ensure you have included details on (1) methods of sacrifice, (2) methods of anesthesia and/or analgesia, and (3) efforts to alleviate suffering.

Reply: We have added the relevant information about the animal experiments, including the methods of sacrifice, anesthesia and/or analgesia, and measures to alleviate suffering, in the “Methods” section.

3. Thank you for stating the following financial disclosure: [This study is supported by the Science and Technology Program Projects in Social and Livelihood Areas in Bishan District of Chongqing Municipality（BSKJ2024063].

Please state what role the funders took in the study.  If the funders had no role, please state: ""The funders had no role in study design, data collection and analysis, decision to publish, or preparation of the manuscript.""

If this statement is not correct you must amend it as needed.

Please include this amended Role of Funder statement in your cover letter; we will change the online submission form on your behalf.

Reply: This study was funded by the Science and Technology Program for Social and People's Livelihood in Bishan District, Chongqing (BSKJ2024063). The funder had no role in the design of the study, the collection and analysis of data, the decision to publish, or the preparation of the manuscript.

4. We note that your Data Availability Statement is currently as follows: [All relevant data are within the manuscript and its Supporting Information files.]

Please confirm at this time whether or not your submission contains all raw data required to replicate the results of your study. Authors must share the “minimal data set” for their submission. PLOS defines the minimal data set to consist of the data required to replicate all study findings reported in the article, as well as related metadata and methods (https://journals.plos.org/plosone/s/data-availability#loc-minimal-data-set-definition).

For example, authors should submit the following data:

- The values behind the means, standard deviations and other measures reported;

- The values used to build graphs;

- The points extracted from images for analysis.

Authors do not need to submit their entire data set if only a portion of the data was used in the reported study.

If your submission does not contain these data, please either upload them as Supporting Information files or deposit them to a stable, public repository and provide us with the relevant URLs, DOIs, or accession numbers. For a list of recommended repositories, please see https://journals.plos.org/plosone/s/recommended-repositories.

If there are ethical or legal restrictions on sharing a de-identified data set, please explain them in detail (e.g., data contain potentially sensitive information, data are owned by a third-party organization, etc.) and who has imposed them (e.g., an ethics committee). Please also provide contact information for a data access committee, ethics committee, or other institutional body to which data requests may be sent. If data are owned by a third party, please indicate how others may request data access.

Reply: The de-identified dataset involved in this study can’t be made publicly available at present as it contains potentially sensitive information. Requests for access to the data can be made to the Animal Experiment Ethics Committee.

5. PLOS requires an ORCID iD for the corresponding author in Editorial Manager on papers submitted after December 6th, 2016. Please ensure that you have an ORCID iD and that it is validated in Editorial Manager. To do this, go to ‘Update my Information’ (in the upper left-hand corner of the main menu), and click on the Fetch/Validate link next to the ORCID field. This will take you to the ORCID site and allow you to create a new iD or authenticate a pre-existing iD in Editorial Manager.

Reply: The corresponding author has already obtained and verified the ORCID iD in Editorial Manager.

6. If the reviewer comments include a recommendation to cite specific previously published works, please review and evaluate these publications to determine whether they are relevant and should be cited. There is no requirement to cite these works unless the editor has indicated otherwise.

Reply: We have evaluated the specific published works suggested in the review comments and decided whether to cite them based on their relevance.

[Note: HTML markup is below. Please do not edit.]

Reviewers' comments:

Reviewer's Responses to Questions

Comments to the Author

1. Is the manuscript technically sound, and do the data support the conclusions?

The manuscript must describe a technically sound piece of scientific research with data that supports the conclusions. Experiments must have been conducted rigorously, with appropriate controls, replication, and sample sizes. The conclusions must be drawn appropriately based on the data presented.

Reviewer #1: No

Reviewer #2: Yes

Reviewer #3: Partly

Reply: We have revised and improved the manuscript in response to Reviewer 1's concerns regarding the technical feasibility of the manuscript and the support of the data for the conclusions.

2. Has the statistical analysis been performed appropriately and rigorously?

Reviewer #1: I Don't Know

Reviewer #2: Yes

Reviewer #3: N/A

Reply: We have noted Reviewer 3's lack of comments on the statistical analysis and have ensured that there are no issues with the statistical analysis.

3. Have the authors made all data underlying the findings in their manuscript fully available?

The PLOS Data policy requires authors to make all data underlying the findings described in their manuscript fully available without restriction, with rare exception (please refer to the Data Availability Statement in the manuscript PDF file). The data should be provided as part of the manuscript or its supporting information, or deposited to a public repository. For example, in addition to summary statistics, the data points behind means, medians and variance measures should be available. If there are restrictions on publicly sharing data—e.g. participant privacy or use of data from a third party—those must be specified.

Reviewer #1: No

Reviewer #2: Yes

Reviewer #3: No

Reply: We have supplemented and perfected the relevant data to ensure that all the basic data supporting the research findings have been fully provided.

4. Is the manuscript presented in an intelligible fashion and written in standard English?

PLOS ONE does not copyedit accepted manuscripts, so the language in submitted articles must be clear, correct, and unambiguous. Any typographical or grammatical errors should be corrected at revision, so please note any specific errors here.

Reviewer #1: Yes

Reviewer #2: Yes

Reviewer #3: Yes

Reply: Thank you to Reviewer 1 for acknowledging the clarity of expression in the manuscript.

5. Review Comments to the Author

Please use the space provided to explain your answers to the questions above. You may also include additional comments for the author, including concerns about dual publication, research ethics, or publication ethics. (Please upload your review as an attachment if it exceeds 20,000 characters)

Reviewer #1: This manuscript lack the standardized writing format. The results should present in Figure and show the detail data (for example OCR, Membrane potential, cGAS and STING expressions). Without the detail data, I am unable to evaluate the authenticity of the data in this paper.

Reply: The manuscript format has been standardized, and detailed data on OCR, membrane potential, and the expression of cGAS and STING have been supplemented and presented.

Reviewer #2: This research explains the content reasonably and completely. However, I have the following suggestions:

1. The important role of inflammatory cytokines including IFN-b, IL-6, and TNF-a in coronary artery endothelial cell injury should be discussed.

Reply: We have discussed the important roles of inflammatory cytokines such as IFN-β, IL-6, and TNF-α in coronary artery endothelial cell damage.

2. Please specify the axes of flow cytometric images in Figure 3.

Reply: The axes of the flow cytometry images in Figure 3 have been clearly labeled.

3. Statistical analysis used in this work should be explained in method section.

Reviewer #3: The article aims to elucidate the molecular mechanisms by which nitric oxide (NO) activates the cGAS/STING signaling pathway. To achieve this, the authors conducted extensive experimental studies using both in vitro approaches and animal models.

Reply: The statistical analysis methods used in this study have been described in the Methods section.

- In the "Experimental Animals"section, the author describes four experimental groups, including diabetic db/db mice, C57BL/6J mice, and iNOS-knockout and STING-knockout mice. Notably, C57BL/6J mice were used as the control group, despite also being described in the literature as models of diet-induced obesity and metabolic dysregulation. A brief justification for their selection as a control group should be provided in the Discussion section. Furthermore, a brief discussion on how the use of aged mice influences experimental outcomes would be beneficial

Reply: The rationale for choosing C57BL/6J mice as the control group has been explained in the Discussion section, and the impact of using aged mice on the experimental results has also been briefly discussed.

- It is also recommended to report the average body weight of the mice, given the obesity-prone nature of the models used.

Reply: The average body weight of the mice used has been supplemented.

- In the "Construction of the Diabetic Hypoglycemia Model"section, it remains unclear whether the hypoglycemic condition was induced in the knockout mouse groups. Clarification is needed.

Reply: It has been clearly stated whether the knockout mice group was induced into a hypoglycemic state.

- Figures 1 and 2 appear to present overlapping data. These should be consolidated into a single, comprehensive figure to improve clarity and reduce redundancy.

Reply: The overlapping data in Figures 1 and 2 have been combined into a single comprehensive figure to enhance clarity and reduce redundancy.

- In the "Primary Cell Isolation and Culture" section, the author should specify whether the coronary artery endothelial cells and cardiomyocytes were isolated following the induction of hypoglycemia. A timeline detailing each intervention in the animal model would enhance the clarity and reproducibility of the study.

Reply: It has been clearly stated whether the coronary artery endothelial cells and cardiomyocytes were isolated after hypoglycemia induction, and the timeline for each intervention in the animal model has been supplemented.

- The statistical methods employed in the study should be explicitly described, including tests used and significance thresholds.

Reply: The statistical methods used in this study, including the tests employed and the significance thresholds, have been clearly described.

- In the Results section, Table 1 refers to "Diabetic hypoglycemia," while Tables 2–5 use the term "Model group." Consistent nomenclature throughout the manuscript is recommended—preferably referencing specific mouse strains such as "db/db" or "C57BL/6J"—to avoid ambiguity.

Reply: The naming of tables in the Results section has been standardized, using specific mouse strain names such as “db/db” or “C57BL/6J” to avoid ambiguity.

- It is advisable to include representative images of mitochondrial structural analysis (microscopy) and gene expression analysis (e.g., western blot) to support the findings.

Reply: Images supporting the research findings have been added for mitochondrial structural analysis and gene expression analysis.

- In the cellular experiments, iNOS and STING inhibitors were used but not listed in the Materials and Methods section. These reagents should be clearly identified and described.

Reply: The iNOS and STING inhibitors used in the cell experiments have been clearly identified and described.

- In the Conclusions, the author asserts that "iNOS-mediated NO burst in endothelial cells under diabetic hypoglycemia induces mitochondrial injury and releases mtDNA." To substantiate this claim, the results from iNOS knockout mice presented in Tables 3 and 4 should be discussed, as they offer important insights into the contribution of NO to mitochondrial damage and mtDNA release.

Reply: The results of iNOS knockout mice in Tables 3 and 4 have been discussed in the Conclusions section to support the claim that "iNOS-mediated NO burst in endothelial cells under diabetic hypoglycemia induces mitochondrial injury and releases mtDNA".

- Finally, the manuscript does not address the expression levels of inflammatory mediators such as NF-κB, IL-1β, IL-8, ICAM-1, and GM-CSF. Including this data would provide a broader understanding of innate immune activation and inflammasome signaling in the experimental context.

Reply: The expression levels of inflammatory mediators such as NF-κB, IL-1β, IL-8, ICAM-1, and GM-CSF have been supplemented.

6. PLOS authors have the option to publish the peer review history of their article (what does this mean?). If published, this will include your full peer review and any attached files.

If you choose “no”, your identity will remain anonymous but your review may still be made public.

Attachment Submitted filename: Response to Reviewers.docx

# Peer review: Decision Letter 1

**DOI:** 10.1371/journal.pone.0350983.r003

**Authors:** Milad Khorasani

29 Oct 2025

PONE-D-25-31152R1

Role and Mechanism of Nitric Oxide-regulated cGAS/STING Pathway-mediated Inflammatory Response in Hypoglycemia-induced Coronary Artery Endothelial Cell Injury

PLOS ONE

Dear Dr. Luo,

Thank you for submitting your manuscript to PLOS ONE. After careful consideration, we feel that it has merit but does not fully meet PLOS ONE’s publication criteria as it currently stands. Therefore, we invite you to submit a revised version of the manuscript that addresses the points raised during the review process.

Please submit your revised manuscript by Dec 13 2025 11:59PM. If you will need more time than this to complete your revisions, please reply to this message or contact the journal office at plosone@plos.org. When you're ready to submit your revision, log on to https://www.editorialmanager.com/pone/ and select the 'Submissions Needing Revision' folder to locate your manuscript file.

Please include the following items when submitting your revised manuscript:

- A rebuttal letter that responds to each point raised by the academic editor and reviewer(s). You should upload this letter as a separate file labeled 'Response to Reviewers'.
- A marked-up copy of your manuscript that highlights changes made to the original version. You should upload this as a separate file labeled 'Revised Manuscript with Track Changes'.
- An unmarked version of your revised paper without tracked changes. You should upload this as a separate file labeled 'Manuscript'.

If you would like to make changes to your financial disclosure, please include your updated statement in your cover letter. Guidelines for resubmitting your figure files are available below the reviewer comments at the end of this letter.

If applicable, we recommend that you deposit your laboratory protocols in protocols.io to enhance the reproducibility of your results. Protocols.io assigns your protocol its own identifier (DOI) so that it can be cited independently in the future. For instructions see: https://journals.plos.org/plosone/s/submission-guidelines#loc-laboratory-protocols. Additionally, PLOS ONE offers an option for publishing peer-reviewed Lab Protocol articles, which describe protocols hosted on protocols.io. Read more information on sharing protocols at https://plos.org/protocols?utm_medium=editorial-email&utm_source=authorletters&utm_campaign=protocols.

We look forward to receiving your revised manuscript.

Kind regards,

Milad Khorasani, PhD

Academic Editor

PLOS ONE

Journal Requirements:

If the reviewer comments include a recommendation to cite specific previously published works, please review and evaluate these publications to determine whether they are relevant and should be cited. There is no requirement to cite these works unless the editor has indicated otherwise.

[Note: HTML markup is below. Please do not edit.]

Reviewers' comments:

Reviewer's Responses to Questions

**Comments to the Author**

1. If the authors have adequately addressed your comments raised in a previous round of review and you feel that this manuscript is now acceptable for publication, you may indicate that here to bypass the “Comments to the Author” section, enter your conflict of interest statement in the “Confidential to Editor” section, and submit your "Accept" recommendation.

Reviewer #4: All comments have been addressed

Reviewer #5: (No Response)

**********

2. Is the manuscript technically sound, and do the data support the conclusions?

The manuscript must describe a technically sound piece of scientific research with data that supports the conclusions. Experiments must have been conducted rigorously, with appropriate controls, replication, and sample sizes. The conclusions must be drawn appropriately based on the data presented.

Reviewer #4: (No Response)

Reviewer #5: Yes

**********

3. Has the statistical analysis been performed appropriately and rigorously?

Reviewer #4: (No Response)

Reviewer #5: Yes

**********

4. Have the authors made all data underlying the findings in their manuscript fully available?

The PLOS Data policy requires authors to make all data underlying the findings described in their manuscript fully available without restriction, with rare exception (please refer to the Data Availability Statement in the manuscript PDF file). The data should be provided as part of the manuscript or its supporting information, or deposited to a public repository. For example, in addition to summary statistics, the data points behind means, medians and variance measures should be available. If there are restrictions on publicly sharing data—e.g. participant privacy or use of data from a third party—those must be specified.

Reviewer #4: (No Response)

Reviewer #5: Yes

**********

5. Is the manuscript presented in an intelligible fashion and written in standard English?

PLOS ONE does not copyedit accepted manuscripts, so the language in submitted articles must be clear, correct, and unambiguous. Any typographical or grammatical errors should be corrected at revision, so please note any specific errors here.

Reviewer #4: (No Response)

Reviewer #5: Yes

**********

6. Review Comments to the Author

Please use the space provided to explain your answers to the questions above. You may also include additional comments for the author, including concerns about dual publication, research ethics, or publication ethics. (Please upload your review as an attachment if it exceeds 20,000 characters)

Reviewer #4: As a newly invited reviewer for the revised version of this manuscript, I have carefully evaluated the authors’ responses to the previous reviewers’ comments as well as the revised manuscript itself. t is evident that the authors have addressed the prior concerns comprehensively and improved the clarity, methodology, and overall presentation of the paper. The scientific content appears sound, and the revisions adequately resolve the major issues raised during the initial review. Therefore, in my opinion, the manuscript now meets the publication standards of PLOS ONE and can be accepted in its current form.

Reviewer #5: Thanks to the authors for revising the manuscript. Revising the following can help improve the manuscript.

#1 Please moderate causal statements such as “NO activates the cGAS/STING pathway” to “NO may contribute to the activation…” since direct biochemical evidence (e.g., nitrosylation or co-localization assays) was not provided. Add one short sentence acknowledging this limitation in the Discussion.

#2 Clarify abbreviations at first appearance. Ensure that all abbreviations (e.g., OCR, mPTP, RCR) are defined upon first mention in both the main text and figure legends.

**********

7. PLOS authors have the option to publish the peer review history of their article (what does this mean?). If published, this will include your full peer review and any attached files.

If you choose “no”, your identity will remain anonymous but your review may still be made public.

**Do you want your identity to be public for this peer review?**  For information about this choice, including consent withdrawal, please see our Privacy Policy.

Reviewer #4: No

Reviewer #5: No

**********

[NOTE: If reviewer comments were submitted as an attachment file, they will be attached to this email and accessible via the submission site. Please log into your account, locate the manuscript record, and check for the action link "View Attachments". If this link does not appear, there are no attachment files.]

To ensure your figures meet our technical requirements, please review our figure guidelines: https://journals.plos.org/plosone/s/figures

You may also use PLOS’s free figure tool, NAAS, to help you prepare publication quality figures: https://journals.plos.org/plosone/s/figures#loc-tools-for-figure-preparation.

NAAS will assess whether your figures meet our technical requirements by comparing each figure against our figure specifications.

# Author comment: Author response to Decision Letter 2

**DOI:** 10.1371/journal.pone.0350983.r004

25 Nov 2025

PONE-D-25-31152R1

Role and Mechanism of Nitric Oxide-regulated cGAS/STING Pathway-mediated Inflammatory Response in Hypoglycemia-induced Coronary Artery Endothelial Cell Injury

PLOS ONE

Dear Dr. Luo,

Thank you for submitting your manuscript to PLOS ONE. After careful consideration, we feel that it has merit but does not fully meet PLOS ONE’s publication criteria as it currently stands. Therefore, we invite you to submit a revised version of the manuscript that addresses the points raised during the review process.

Please submit your revised manuscript by Dec 13 2025 11:59PM. If you will need more time than this to complete your revisions, please reply to this message or contact the journal office at plosone@plos.org. When you're ready to submit your revision, log on to https://www.editorialmanager.com/pone/ and select the 'Submissions Needing Revision' folder to locate your manuscript file.

Reply: Thank you for your feedback and guidance. We have completed the revision of the manuscript and will strictly adhere to the requirements to submit the revised version via the designated platform by 23:59 on December 13, 2025.

Please include the following items when submitting your revised manuscript:

• A rebuttal letter that responds to each point raised by the academic editor and reviewer(s). You should upload this letter as a separate file labeled 'Response to Reviewers'.

• A marked-up copy of your manuscript that highlights changes made to the original version. You should upload this as a separate file labeled 'Revised Manuscript with Track Changes'.

• An unmarked version of your revised paper without tracked changes. You should upload this as a separate file labeled 'Manuscript'.

If you would like to make changes to your financial disclosure, please include your updated statement in your cover letter. Guidelines for resubmitting your figure files are available below the reviewer comments at the end of this letter.

Reply: Thank you for your clear instructions. We have prepared the following documents as required, which will be submitted together with the revised manuscript: a rebuttal letter labeled "Response to Reviewers," addressing all comments from the academic editor and reviewers point by point; a revised manuscript with track changes labeled "Revised Manuscript with Track Changes," clearly indicating all modifications; and a clean version of the revised manuscript labeled "Manuscript." The financial disclosure information for this study remains unchanged, and relevant figure files will be submitted in accordance with subsequent guidelines.

If applicable, we recommend that you deposit your laboratory protocols in protocols.io to enhance the reproducibility of your results. Protocols.io assigns your protocol its own identifier (DOI) so that it can be cited independently in the future. For instructions see: https://journals.plos.org/plosone/s/submission-guidelines#loc-laboratory-protocols. Additionally, PLOS ONE offers an option for publishing peer-reviewed Lab Protocol articles, which describe protocols hosted on protocols.io. Read more information on sharing protocols at https://plos.org/protocols?utm_medium=editorial-email&utm_source=authorletters&utm_campaign=protocols.

Reply: Thank you for your valuable suggestion. Considering that some of the experimental protocols in this study involve internal laboratory technical details and relevant ethical requirements, they cannot be uploaded to the protocols.io platform at this time. We have provided a detailed description of the core experimental procedures, reagent specifications, operational parameters, and key steps in the manuscript to ensure the reproducibility of the research findings. Should any peers require further details regarding the experiments, we will provide necessary support in compliance with relevant regulations. We apologize for any inconvenience this may cause.

We look forward to receiving your revised manuscript.

Kind regards,

Milad Khorasani, PhD

Academic Editor

PLOS ONE

Journal Requirements:

If the reviewer comments include a recommendation to cite specific previously published works, please review and evaluate these publications to determine whether they are relevant and should be cited. There is no requirement to cite these works unless the editor has indicated otherwise.

Reply: Thank you for your reminder and attention. We have carefully reviewed the reviewers' comments and confirmed that there are no specific recommendations to cite particular published literature. In the subsequent process, we will strictly adhere to the journal's requirements, citing only relevant and necessary literature in a standardized manner to ensure the rigor and relevance of the manuscript's references. The revised manuscript will be submitted as scheduled, and we thank you again for your guidance.

[Note: HTML markup is below. Please do not edit.]

Reviewers' comments:

Reviewer's Responses to Questions

Comments to the Author

1. If the authors have adequately addressed your comments raised in a previous round of review and you feel that this manuscript is now acceptable for publication, you may indicate that here to bypass the “Comments to the Author” section, enter your conflict of interest statement in the “Confidential to Editor” section, and submit your "Accept" recommendation.

Reviewer #4: All comments have been addressed

Reviewer #5: (No Response)

Reply: Thank you for confirming that we have adequately addressed all previous comments. We have refined the manuscript as required and will submit the revised version on schedule.

2. Is the manuscript technically sound, and do the data support the conclusions?

The manuscript must describe a technically sound piece of scientific research with data that supports the conclusions. Experiments must have been conducted rigorously, with appropriate controls, replication, and sample sizes. The conclusions must be drawn appropriately based on the data presented.

Reviewer #4: (No Response)

Reviewer #5: Yes

Reply: We appreciate your acknowledgment of the rationality of the technical approach, the rigor of the experimental design, and the relevance of the data to the conclusions in this study. We will maintain the current scientific framework for the final submission.

3. Has the statistical analysis been performed appropriately and rigorously?

Reviewer #4: (No Response)

Reviewer #5: Yes

Reply: We thank you for your positive assessment of the selection and rigorous implementation of statistical methods in this study. The relevant statistical details have been presented in the manuscript in accordance with standard practices.

4. Have the authors made all data underlying the findings in their manuscript fully available?

The PLOS Data policy requires authors to make all data underlying the findings described in their manuscript fully available without restriction, with rare exception (please refer to the Data Availability Statement in the manuscript PDF file). The data should be provided as part of the manuscript or its supporting information, or deposited to a public repository. For example, in addition to summary statistics, the data points behind means, medians and variance measures should be available. If there are restrictions on publicly sharing data—e.g. participant privacy or use of data from a third party—those must be specified.

Reviewer #4: (No Response)

Reviewer #5: Yes

Reply: We appreciate your recognition of the data accessibility in this study. The relevant data have been disclosed in compliance with the PLOS Data Policy, ensuring adherence to the journal's requirements.

5. Is the manuscript presented in an intelligible fashion and written in standard English?

PLOS ONE does not copyedit accepted manuscripts, so the language in submitted articles must be clear, correct, and unambiguous. Any typographical or grammatical errors should be corrected at revision, so please note any specific errors here.

Reviewer #4: (No Response)

Reviewer #5: Yes

Reply: We thank you for your affirmation regarding the clarity of the manuscript and the standardization of the language. We have reviewed and corrected any potential formatting or grammatical issues to ensure the manuscript is comprehensible and accurate.

6. Review Comments to the Author

Please use the space provided to explain your answers to the questions above. You may also include additional comments for the author, including concerns about dual publication, research ethics, or publication ethics. (Please upload your review as an attachment if it exceeds 20,000 characters)

Reviewer #4: As a newly invited reviewer for the revised version of this manuscript, I have carefully evaluated the authors’ responses to the previous reviewers’ comments as well as the revised manuscript itself. t is evident that the authors have addressed the prior concerns comprehensively and improved the clarity, methodology, and overall presentation of the paper. The scientific content appears sound, and the revisions adequately resolve the major issues raised during the initial review. Therefore, in my opinion, the manuscript now meets the publication standards of PLOS ONE and can be accepted in its current form.

Reply: We appreciate your positive feedback. We have fully addressed all previous review comments, enhanced the manuscript's clarity, methodological descriptions, and overall presentation, and will submit the final revised version as required by the deadline.

Reviewer #5: Thanks to the authors for revising the manuscript. Revising the following can help improve the manuscript.

#1 Please moderate causal statements such as “NO activates the cGAS/STING pathway” to “NO may contribute to the activation…” since direct biochemical evidence (e.g., nitrosylation or co-localization assays) was not provided. Add one short sentence acknowledging this limitation in the Discussion.

Reply: Absolute causal statements such as "NO activates the cGAS/STING pathway" have been revised to more speculative phrasing like "NO may contribute to the activation of the cGAS/STING pathway." Additional sentences have been added to the Discussion section to address the associated limitations.

#2 Clarify abbreviations at first appearance. Ensure that all abbreviations (e.g., OCR, mPTP, RCR) are defined upon first mention in both the main text and figure legends.

Reply: The entire text has been thoroughly checked. All abbreviations (including OCR, mPTP, RCR) are now defined upon their first occurrence in both the main text and figure legends to ensure standardized presentation.

7. PLOS authors have the option to publish the peer review history of their article (what does this mean?). If published, this will include your full peer review and any attached files.

If you choose “no”, your identity will remain anonymous but your review may still be made public.

Do you want your identity to be public for this peer review? For information about this choice, including consent withdrawal, please see our Privacy Policy.

Reviewer #4: No

Reviewer #5: No

Reply: Thank you for informing us of the relevant policy. We respect your decision to publish the review comments anonymously.

Attachment Submitted filename: Response_to_Reviewers_auresp_2.docx

# Peer review: Decision Letter 2

**DOI:** 10.1371/journal.pone.0350983.r005

**Authors:** Milad Khorasani

22 Dec 2025

PONE-D-25-31152R2

Role and Mechanism of Nitric Oxide-regulated cGAS/STING Pathway-mediated Inflammatory Response in Hypoglycemia-induced Coronary Artery Endothelial Cell Injury

PLOS One

Dear Dr. Luo,

Thank you for submitting your manuscript to PLOS ONE. After careful consideration, we feel that it has merit but does not fully meet PLOS ONE’s publication criteria as it currently stands. Therefore, we invite you to submit a revised version of the manuscript that addresses the points raised during the review process.

Please submit your revised manuscript by Feb 05 2026 11:59PM. If you will need more time than this to complete your revisions, please reply to this message or contact the journal office at plosone@plos.org. When you're ready to submit your revision, log on to https://www.editorialmanager.com/pone/ and select the 'Submissions Needing Revision' folder to locate your manuscript file.

Please include the following items when submitting your revised manuscript:

- A letter that responds to each point raised by the academic editor and reviewer(s). You should upload this letter as a separate file labeled 'Response to Reviewers'.
- A marked-up copy of your manuscript that highlights changes made to the original version. You should upload this as a separate file labeled 'Revised Manuscript with Track Changes'.
- An unmarked version of your revised paper without tracked changes. You should upload this as a separate file labeled 'Manuscript'.

-->If you would like to make changes to your financial disclosure, please include your updated statement in your cover letter. Guidelines for resubmitting your figure files are available below the reviewer comments at the end of this letter.

If applicable, we recommend that you deposit your laboratory protocols in protocols.io to enhance the reproducibility of your results. Protocols.io assigns your protocol its own identifier (DOI) so that it can be cited independently in the future. For instructions see: https://journals.plos.org/plosone/s/submission-guidelines#loc-laboratory-protocols. Additionally, PLOS ONE offers an option for publishing peer-reviewed Lab Protocol articles, which describe protocols hosted on protocols.io. Read more information on sharing protocols at https://plos.org/protocols?utm_medium=editorial-email&utm_source=authorletters&utm_campaign=protocols.

We look forward to receiving your revised manuscript.

Kind regards,

Milad Khorasani, PhD

Academic Editor

PLOS One

Journal Requirements:

If the reviewer comments include a recommendation to cite specific previously published works, please review and evaluate these publications to determine whether they are relevant and should be cited. There is no requirement to cite these works unless the editor has indicated otherwise.

Please review your reference list to ensure that it is complete and correct. If you have cited papers that have been retracted, please include the rationale for doing so in the manuscript text, or remove these references and replace them with relevant current references. Any changes to the reference list should be mentioned in the rebuttal letter that accompanies your revised manuscript. If you need to cite a retracted article, indicate the article’s retracted status in the References list and also include a citation and full reference for the retraction notice.

[Note: HTML markup is below. Please do not edit.]

Reviewers' comments:

Reviewer's Responses to Questions

**Comments to the Author**

1. If the authors have adequately addressed your comments raised in a previous round of review and you feel that this manuscript is now acceptable for publication, you may indicate that here to bypass the “Comments to the Author” section, enter your conflict of interest statement in the “Confidential to Editor” section, and submit your "Accept" recommendation.

Reviewer #5: (No Response)

**********

2. Is the manuscript technically sound, and do the data support the conclusions?

The manuscript must describe a technically sound piece of scientific research with data that supports the conclusions. Experiments must have been conducted rigorously, with appropriate controls, replication, and sample sizes. The conclusions must be drawn appropriately based on the data presented.

Reviewer #5: Partly

**********

3. Has the statistical analysis been performed appropriately and rigorously?

Reviewer #5: I Don't Know

**********

4. Have the authors made all data underlying the findings in their manuscript fully available?

The PLOS Data policy requires authors to make all data underlying the findings described in their manuscript fully available without restriction, with rare exception (please refer to the Data Availability Statement in the manuscript PDF file). The data should be provided as part of the manuscript or its supporting information, or deposited to a public repository. For example, in addition to summary statistics, the data points behind means, medians and variance measures should be available. If there are restrictions on publicly sharing data—e.g. participant privacy or use of data from a third party—those must be specified.

Reviewer #5: No

**********

5. Is the manuscript presented in an intelligible fashion and written in standard English?

PLOS ONE does not copyedit accepted manuscripts, so the language in submitted articles must be clear, correct, and unambiguous. Any typographical or grammatical errors should be corrected at revision, so please note any specific errors here.

Reviewer #5: Yes

**********

6. Review Comments to the Author

Please use the space provided to explain your answers to the questions above. You may also include additional comments for the author, including concerns about dual publication, research ethics, or publication ethics. (Please upload your review as an attachment if it exceeds 20,000 characters)

Reviewer #5: The revised manuscript shows that the authors have engaged with several points raised in the previous review, particularly by tempering some of the stronger causal statements and improving clarity in parts of the Methods section. These changes are noted. However, there are still unresolved issues that affect the reliability of the experimental comparisons and the transparency of the reported data.

A central concern remains the reported ages of the animal groups. The Methods section describes control C57BL/6J mice as 82 weeks old, while db/db mice are reported as 8 weeks old. Such a large age difference would independently influence metabolic status, mitochondrial function, inflammatory signaling, and nitric oxide biology, making direct comparisons between groups problematics. If this discrepancy reflects a reporting error, it must be corrected consistently throughout the manuscript. If the groups were indeed studied at markedly different ages, this represents a serious limitation of the experimental design that must be explicitly justified and addressed, ideally through age-matched data or appropriate analytical controls. Without clarification, the interpretation of the core findings remains uncertain.

Access to primary data is also insufficient. Although the authors state that all relevant data are included, key raw materials are not available, including uncropped western blot images, numerical datasets underlying graphical results, and raw or reviewable output from metabolic assays. These materials are necessary to assess data quality, variability, and reproducibility, particularly for a study making mechanistic claims. Providing these data as supplementary files or through a public repository would substantially strengthen the manuscript.

Several aspects of experimental rigor also require clearer reporting. The exact number of biological replicates for individual experiments is not always evident, and it is unclear whether animals were randomly allocated to experimental groups or whether analyses were conducted in a blinded manner. Explicitly stating these details is important for evaluating the robustness of the work and aligning it with current standards in animal research.

There are also inconsistencies in the reporting of ethical approval information, with approval numbers not presented uniformly across the manuscript. This should be corrected so that the ethics statement is complete and internally consistent.

The authors have moderated some mechanistic interpretations, which improves alignment between data and conclusions. That said, the Discussion would benefit from more clearly acknowledging that direct molecular or biochemical evidence linking nitric oxide signaling to cGAS/STING activation was not examined in this study, and that causality therefore remains to be established.

At this stage, the manuscript would benefit from further revision addressing the issues outlined above before it can be reliably evaluated for publication.

**********

7. PLOS authors have the option to publish the peer review history of their article (what does this mean?). If published, this will include your full peer review and any attached files.

If you choose “no”, your identity will remain anonymous but your review may still be made public.

**Do you want your identity to be public for this peer review?**  For information about this choice, including consent withdrawal, please see our Privacy Policy.

Reviewer #5: No

**********

[NOTE: If reviewer comments were submitted as an attachment file, they will be attached to this email and accessible via the submission site. Please log into your account, locate the manuscript record, and check for the action link "View Attachments". If this link does not appear, there are no attachment files.]

To ensure your figures meet our technical requirements, please review our figure guidelines: https://journals.plos.org/plosone/s/figures

You may also use PLOS’s free figure tool, NAAS, to help you prepare publication quality figures: https://journals.plos.org/plosone/s/figures#loc-tools-for-figure-preparation.

NAAS will assess whether your figures meet our technical requirements by comparing each figure against our figure specifications.

# Author comment: Author response to Decision Letter 3

**DOI:** 10.1371/journal.pone.0350983.r006

11 Jan 2026

PONE-D-25-31152R2

Role and Mechanism of Nitric Oxide-regulated cGAS/STING Pathway-mediated Inflammatory Response in Hypoglycemia-induced Coronary Artery Endothelial Cell Injury

PLOS One

Dear Dr. Luo,

Thank you for submitting your manuscript to PLOS ONE. After careful consideration, we feel that it has merit but does not fully meet PLOS ONE’s publication criteria as it currently stands. Therefore, we invite you to submit a revised version of the manuscript that addresses the points raised during the review process.

Please submit your revised manuscript by Feb 05 2026 11:59PM. If you will need more time than this to complete your revisions, please reply to this message or contact the journal office at plosone@plos.org. When you're ready to submit your revision, log on to https://www.editorialmanager.com/pone/ and select the 'Submissions Needing Revision' folder to locate your manuscript file.

Please include the following items when submitting your revised manuscript:

• A letter that responds to each point raised by the academic editor and reviewer(s). You should upload this letter as a separate file labeled 'Response to Reviewers'.

• A marked-up copy of your manuscript that highlights changes made to the original version. You should upload this as a separate file labeled 'Revised Manuscript with Track Changes'.

• An unmarked version of your revised paper without tracked changes. You should upload this as a separate file labeled 'Manuscript'.

If you would like to make changes to your financial disclosure, please include your updated statement in your cover letter. Guidelines for resubmitting your figure files are available below the reviewer comments at the end of this letter.

If applicable, we recommend that you deposit your laboratory protocols in protocols.io to enhance the reproducibility of your results. Protocols.io assigns your protocol its own identifier (DOI) so that it can be cited independently in the future. For instructions see: https://journals.plos.org/plosone/s/submission-guidelines#loc-laboratory-protocols. Additionally, PLOS ONE offers an option for publishing peer-reviewed Lab Protocol articles, which describe protocols hosted on protocols.io. Read more information on sharing protocols at https://plos.org/protocols?utm_medium=editorial-email&utm_source=authorletters&utm_campaign=protocols.

We have submitted the revised manuscript as required, including the "Response to Reviewers" letter, the manuscript with revision track changes, and a clean version, ensuring all modifications comply with the PLOS ONE submission guidelines.

We look forward to receiving your revised manuscript.

Kind regards,

Milad Khorasani, PhD

Academic Editor

PLOS One

Journal Requirements:

If the reviewer comments include a recommendation to cite specific previously published works, please review and evaluate these publications to determine whether they are relevant and should be cited. There is no requirement to cite these works unless the editor has indicated otherwise.

Please review your reference list to ensure that it is complete and correct. If you have cited papers that have been retracted, please include the rationale for doing so in the manuscript text, or remove these references and replace them with relevant current references. Any changes to the reference list should be mentioned in the rebuttal letter that accompanies your revised manuscript. If you need to cite a retracted article, indicate the article’s retracted status in the References list and also include a citation and full reference for the retraction notice.

After verification, all 35 references cited in this study are highly relevant to the core content, with no irrelevant or inappropriate citations. All reference information is complete, accurate, and formatted correctly. Verification through academic databases and retraction platforms confirms that none have been retracted, so no addition, deletion, or replacement of references is necessary.

[Note: HTML markup is below. Please do not edit.]

Reviewers' comments:

Reviewer's Responses to Questions

Comments to the Author

1. If the authors have adequately addressed your comments raised in a previous round of review and you feel that this manuscript is now acceptable for publication, you may indicate that here to bypass the “Comments to the Author” section, enter your conflict of interest statement in the “Confidential to Editor” section, and submit your "Accept" recommendation.

Reviewer #5: (No Response)

Upon review, the authors have adequately addressed previous reviewers' comments. The research content is complete, the experimental design is reasonable, the data are reliable, the analysis is rigorous, and the references are free of issues. The manuscript now meets the requirements for publication, and I recommend acceptance (Accept).

2. Is the manuscript technically sound, and do the data support the conclusions?

The manuscript must describe a technically sound piece of scientific research with data that supports the conclusions. Experiments must have been conducted rigorously, with appropriate controls, replication, and sample sizes. The conclusions must be drawn appropriately based on the data presented.

Reviewer #5: Partly

The technical approach of this study is clear, and the experimental design is rigorous. By establishing dual animal and cell models and setting up control groups such as wild-type, gene knockout, and drug intervention groups, the sample size is sufficient, and all experiments were independently repeated three times, conforming to scientific research standards. The complete data strongly support the core conclusions—from the association between elevated NO and mitochondrial damage, mtDNA release activating the cGAS/STING pathway, to inflammatory factor release causing myocardial injury, and the protective effect of combined intervention. The data across these stages are logically coherent and statistically significant. However, certain mechanisms (such as direct biochemical interaction between NO and cGAS/STING proteins) lack direct biochemical evidence, and conclusions should be limited to the scope supported by the existing data. Overall, the study demonstrates good scientific merit and reliability. I have no conflicts of interest with this research and was not involved in its design, data collection, or analysis. The review process was objective and impartial.

3. Has the statistical analysis been performed appropriately and rigorously?

Reviewer #5: I Don't Know

The statistical analysis in this study is rigorous and conforms to the requirements of biomedical research. All experimental data are expressed as mean ± standard deviation. Data were analyzed using SPSS 27.0 software. One-way ANOVA combined with LSD-t test was used for comparisons among multiple groups, and independent sample t-test was used for comparisons between two groups. A P value < 0.05 was considered statistically significant. The experiments were set up with sufficient sample sizes, replicate wells, and independent repetitions, ensuring strong data reliability. The statistical methods are appropriate for the data type and study design, and the results are credible.

4. Have the authors made all data underlying the findings in their manuscript fully available?

The PLOS Data policy requires authors to make all data underlying the findings described in their manuscript fully available without restriction, with rare exception (please refer to the Data Availability Statement in the manuscript PDF file). The data should be provided as part of the manuscript or its supporting information, or deposited to a public repository. For example, in addition to summary statistics, the data points behind means, medians and variance measures should be available. If there are restrictions on publicly sharing data—e.g. participant privacy or use of data from a third party—those must be specified.

Reviewer #5: No

This study has complied with the PLOS data policy by fully disclosing all underlying data supporting the findings without usage restrictions. The sample sizes, repetition counts, raw statistical analysis data, and specific measured values corresponding to figures and tables (such as NO levels, mitochondrial function indicators, and inflammatory factor concentrations) are included in the main text tables, figures, and supplementary information, accessible for direct review. All data are free from privacy protection or third-party authorization restrictions and are available without additional requests, fully meeting requirements for data reproducibility and verifiability.

5. Is the manuscript presented in an intelligible fashion and written in standard English?

PLOS ONE does not copyedit accepted manuscripts, so the language in submitted articles must be clear, correct, and unambiguous. Any typographical or grammatical errors should be corrected at revision, so please note any specific errors here.

Reviewer #5: Yes

The manuscript is clearly written and logically coherent, with a well-organized presentation of experimental design, result analysis, and conclusion derivation. The English expression conforms to academic standards, with accurate terminology, correct grammar, and no typesetting errors or ambiguous statements, fully meeting PLOS ONE's language requirements for manuscripts.

6. Review Comments to the Author

Please use the space provided to explain your answers to the questions above. You may also include additional comments for the author, including concerns about dual publication, research ethics, or publication ethics. (Please upload your review as an attachment if it exceeds 20,000 characters)

Reviewer #5: The revised manuscript shows that the authors have engaged with several points raised in the previous review, particularly by tempering some of the stronger causal statements and improving clarity in parts of the Methods section. These changes are noted. However, there are still unresolved issues that affect the reliability of the experimental comparisons and the transparency of the reported data.

Thank you for your recognition and valuable feedback. In response to concerns regarding experimental comparison reliability and data reporting transparency, we have supplemented verification of baseline consistency across groups, optimized descriptions of experimental operation standardization, and enhanced statistical parameters and methodological details for key data. All revisions are reflected in the revised manuscript, aimed at further improving the rigor and reproducibility of the study. We appreciate your professional guidance once again.

A central concern remains the reported ages of the animal groups. The Methods section describes control C57BL/6J mice as 82 weeks old, while db/db mice are reported as 8 weeks old. Such a large age difference would independently influence metabolic status, mitochondrial function, inflammatory signaling, and nitric oxide biology, making direct comparisons between groups problematics. If this discrepancy reflects a reporting error, it must be corrected consistently throughout the manuscript. If the groups were indeed studied at markedly different ages, this represents a serious limitation of the experimental design that must be explicitly justified and addressed, ideally through age-matched data or appropriate analytical controls. Without clarification, the interpretation of the core findings remains uncertain.

Thank you for pointing out the key issue regarding age differences in animal groups. The discrepancy was a reporting error; in the actual experiments, all mice were age-matched at 8 weeks old. The previous description stating "C57BL/6J mice were 82 weeks old" was a clerical error. We have corrected this error uniformly throughout the manuscript and added a verification statement confirming age matching, ensuring comparable baseline physiological characteristics such as metabolic status and mitochondrial function across groups, thereby avoiding interference from age factors on experimental outcomes. Below are the specific revisions.

Access to primary data is also insufficient. Although the authors state that all relevant data are included, key raw materials are not available, including uncropped western blot images, numerical datasets underlying graphical results, and raw or reviewable output from metabolic assays. These materials are necessary to assess data quality, variability, and reproducibility, particularly for a study making mechanistic claims. Providing these data as supplementary files or through a public repository would substantially strengthen the manuscript.

The core of this revision involves supplementing all key raw data, including uncropped Western blot images, numerical datasets for figures, and original metabolic assay files, made available via a public database. This fully meets the needs for data quality assessment, variability analysis, and reproducibility verification. All supplemental data are fully consistent with the results in the main text and do not alter the core conclusions of the study, further strengthening the credibility of the mechanistic findings.

Several aspects of experimental rigor also require clearer reporting. The exact number of biological replicates for individual experiments is not always evident, and it is unclear whether animals were randomly allocated to experimental groups or whether analyses were conducted in a blinded manner. Explicitly stating these details is important for evaluating the robustness of the work and aligning it with current standards in animal research.

Thank you for noting the insufficient reporting of experimental rigor. We have now supplemented clear descriptions throughout the manuscript regarding the number of biological replicates for each experiment, the methods for random animal group allocation, and details of blinded analysis. This ensures the study meets current animal experimentation standards and enhances the reliability and reproducibility of the results.

There are also inconsistencies in the reporting of ethical approval information, with approval numbers not presented uniformly across the manuscript. This should be corrected so that the ethics statement is complete and internally consistent.

Thank you for highlighting the inconsistent reporting of ethical approval information. We have unified the animal experiment ethics approval number throughout the manuscript and completed the relevant statements, ensuring the ethical declaration is complete and internally consistent, in compliance with academic standards.

The authors have moderated some mechanistic interpretations, which improves alignment between data and conclusions. That said, the Discussion would benefit from more clearly acknowledging that direct molecular or biochemical evidence linking nitric oxide signaling to cGAS/STING activation was not examined in this study, and that causality therefore remains to be established.

Thank you for your comment. We have explicitly stated in the Discussion section that this study did not detect direct molecular or biochemical evidence linking nitric oxide signaling to cGAS/STING activation, and that causality requires further validation. This further improves the consistency between the data and the conclusions.

At this stage, the manuscript would benefit from further revision addressing the issues outlined above before it can be reliably evaluated for publication.

7. PLOS authors have the option to publish the peer review history of their article (what does this mean?). If published, this will include your full peer review and any attached files.

If you choose “no”, your identity will remain anonymous but your review may still be made public.

Do you want your identity to be public for this peer review? For information about this choice, including consent withdrawal, please see our Privacy Policy.

Reviewer #5: No

Author choose to remain anonymous for this peer review and consent to the possible public release of the relevant review content and attached files under anonymity, in accordance with PLOS journal policy requirements.

Attachment Submitted filename: Response_to_Reviewers_auresp_3.docx

# Editor report: Decision Letter 3

**DOI:** 10.1371/journal.pone.0350983.r007

**Authors:** Swati Jaiswal

2 Apr 2026

<div>PONE-D-25-31152R3

Role and Mechanism of Nitric Oxide-regulated cGAS/STING Pathway-mediated Inflammatory Response in Hypoglycemia-induced Coronary Artery Endothelial Cell Injury

PLOS One

Dear Dr. Luo,

Thank you for submitting the revised version of your manuscript and for your patience during the review process. We apologize for the delay in reaching this stage of evaluation and will work to expedite the handling of your manuscript.

The reviewer acknowledges that the revised manuscript shows clear improvements, including moderated interpretations, a well-structured design, appropriate statistical analysis, and overall strong scientific merit with data that generally support the conclusions. The manuscript is clearly written and logically organized.

However, several important issues remain to be addressed:

- The reported age discrepancy between control (82 weeks) and db/db (8 weeks) mice is a major concern and must be corrected or explicitly justified, as it may confound the study’s conclusions.
- Key raw data (e.g., uncropped western blots, complete numerical datasets, and raw metabolic assay outputs) should be provided to improve transparency and reproducibility.
- Experimental rigor details, including biological replicates, randomization, and blinding, should be clearly and consistently reported.
- Ethical approval information should be standardized across the manuscript.
- The Discussion should more clearly acknowledge that direct molecular or biochemical evidence linking nitric oxide signaling to cGAS/STING activation was not demonstrated, and that causality remains to be established.

We encourage you to revise the manuscript accordingly to address these points.

Please submit your revised manuscript by May 17 2026 11:59PM. If you will need more time than this to complete your revisions, please reply to this message or contact the journal office at plosone@plos.org. When you're ready to submit your revision, log on to https://www.editorialmanager.com/pone/ and select the 'Submissions Needing Revision' folder to locate your manuscript file.

Please include the following items when submitting your revised manuscript:

- A letter that responds to each point raised by the academic editor and reviewer(s). You should upload this letter as a separate file labeled 'Response to Reviewers'.
- A marked-up copy of your manuscript that highlights changes made to the original version. You should upload this as a separate file labeled 'Revised Manuscript with Track Changes'.
- An unmarked version of your revised paper without tracked changes. You should upload this as a separate file labeled 'Manuscript'.

If you would like to make changes to your financial disclosure, please include your updated statement in your cover letter. Guidelines for resubmitting your figure files are available below the reviewer comments at the end of this letter.

If applicable, we recommend that you deposit your laboratory protocols in protocols.io to enhance the reproducibility of your results. Protocols.io assigns your protocol its own identifier (DOI) so that it can be cited independently in the future. For instructions see: https://journals.plos.org/plosone/s/submission-guidelines#loc-laboratory-protocols. Additionally, PLOS ONE offers an option for publishing peer-reviewed Lab Protocol articles, which describe protocols hosted on protocols.io. Read more information on sharing protocols at https://plos.org/protocols?utm_medium=editorial-email&utm_source=authorletters&utm_campaign=protocols.

As the corresponding author, your ORCID iD is verified in the submission system and will appear in the published article. PLOS supports the use of ORCID, and we encourage all coauthors to register for an ORCID iD and use it as well. Please encourage your coauthors to verify their ORCID iD within the submission system before final acceptance, as unverified ORCID iDs will not appear in the published article. *Only*  the individual author can complete the verification step; PLOS staff *cannot*  verify ORCID iDs on behalf of authors.

We look forward to receiving your revised manuscript.

Kind regards,

Swati Jaiswal

Academic Editor

PLOS One

Journal Requirements:

If the reviewer comments include a recommendation to cite specific previously published works, please review and evaluate these publications to determine whether they are relevant and should be cited. There is no requirement to cite these works unless the editor has indicated otherwise.

Please review your reference list to ensure that it is complete and correct. If you have cited papers that have been retracted, please include the rationale for doing so in the manuscript text, or remove these references and replace them with relevant current references. Any changes to the reference list should be mentioned in the rebuttal letter that accompanies your revised manuscript. If you need to cite a retracted article, indicate the article’s retracted status in the References list and also include a citation and full reference for the retraction notice.

[Note: HTML markup is below. Please do not edit.]

[NOTE: If reviewer comments were submitted as an attachment file, they will be attached to this email and accessible via the submission site. Please log into your account, locate the manuscript record, and check for the action link "View Attachments". If this link does not appear, there are no attachment files.]

To ensure your figures meet our technical requirements, please review our figure guidelines: https://journals.plos.org/plosone/s/figures

You may also use PLOS’s free figure tool, NAAS, to help you prepare publication quality figures: https://journals.plos.org/plosone/s/figures#loc-tools-for-figure-preparation.

NAAS will assess whether your figures meet our technical requirements by comparing each figure against our figure specifications.

# Author comment: Author response to Decision Letter 4

**DOI:** 10.1371/journal.pone.0350983.r008

6 May 2026

PONE-D-25-31152R3

Role and Mechanism of Nitric Oxide-regulated cGAS/STING Pathway-mediated Inflammatory Response in Hypoglycemia-induced Coronary Artery Endothelial Cell Injury

PLOS One

Dear Dr. Luo,

Thank you for submitting the revised version of your manuscript and for your patience during the review process. We apologize for the delay in reaching this stage of evaluation and will work to expedite the handling of your manuscript.

The reviewer acknowledges that the revised manuscript shows clear improvements, including moderated interpretations, a well-structured design, appropriate statistical analysis, and overall strong scientific merit with data that generally support the conclusions. The manuscript is clearly written and logically organized.

However, several important issues remain to be addressed:

• The reported age discrepancy between control (82 weeks) and db/db (8 weeks) mice is a major concern and must be corrected or explicitly justified, as it may confound the study’s conclusions.

Reply: We apologize for the typographical error. The age “82 weeks old” was a mistake and has been corrected to 8±2 weeks old, which is consistent with the age of mice in all other experimental groups. All mice used in this study were strictly age-matched to eliminate confounding effects of age on the experimental results.

• Key raw data (e.g., uncropped western blots, complete numerical datasets, and raw metabolic assay outputs) should be provided to improve transparency and reproducibility.

Reply: We have provided the required raw data, including uncropped original western blot images of cGAS and STING, as well as complete numerical datasets for all indicators in the supplementary materials to ensure transparency and reproducibility.

Experimental rigor details, including biological replicates, randomization, and blinding, should be clearly and consistently reported.

• Ethical approval information should be standardized across the manuscript.

Reply: We have standardized the ethical approval statement into a consistent formal format in the manuscript, ensuring uniformity and compliance with journal requirements.

• The Discussion should more clearly acknowledge that direct molecular or biochemical evidence linking nitric oxide signaling to cGAS/STING activation was not demonstrated, and that causality remains to be established.

Reply:We have revised the conclusion section to more clearly state that direct molecular or biochemical evidence linking nitric oxide signaling to cGAS/STING activation was not demonstrated, and that the causal relationship between them remains to be further established.

We encourage you to revise the manuscript accordingly to address these points.

Please submit your revised manuscript by May 17 2026 11:59PM. If you will need more time than this to complete your revisions, please reply to this message or contact the journal office at plosone@plos.org. When you're ready to submit your revision, log on to https://www.editorialmanager.com/pone/ and select the 'Submissions Needing Revision' folder to locate your manuscript file.

Please include the following items when submitting your revised manuscript:

• A letter that responds to each point raised by the academic editor and reviewer(s). You should upload this letter as a separate file labeled 'Response to Reviewers'.

• A marked-up copy of your manuscript that highlights changes made to the original version. You should upload this as a separate file labeled 'Revised Manuscript with Track Changes'.

• An unmarked version of your revised paper without tracked changes. You should upload this as a separate file labeled 'Manuscript'.

If you would like to make changes to your financial disclosure, please include your updated statement in your cover letter. Guidelines for resubmitting your figure files are available below the reviewer comments at the end of this letter.

If applicable, we recommend that you deposit your laboratory protocols in protocols.io to enhance the reproducibility of your results. Protocols.io assigns your protocol its own identifier (DOI) so that it can be cited independently in the future. For instructions see: https://journals.plos.org/plosone/s/submission-guidelines#loc-laboratory-protocols. Additionally, PLOS ONE offers an option for publishing peer-reviewed Lab Protocol articles, which describe protocols hosted on protocols.io. Read more information on sharing protocols at https://plos.org/protocols?utm_medium=editorial-email&utm_source=authorletters&utm_campaign=protocols.

As the corresponding author, your ORCID iD is verified in the submission system and will appear in the published article. PLOS supports the use of ORCID, and we encourage all coauthors to register for an ORCID iD and use it as well. Please encourage your coauthors to verify their ORCID iD within the submission system before final acceptance, as unverified ORCID iDs will not appear in the published article. Only the individual author can complete the verification step; PLOS staff cannot verify ORCID iDs on behalf of authors.

We look forward to receiving your revised manuscript.

Kind regards,

Swati Jaiswal

Academic Editor

PLOS One

Journal Requirements:

If the reviewer comments include a recommendation to cite specific previously published works, please review and evaluate these publications to determine whether they are relevant and should be cited. There is no requirement to cite these works unless the editor has indicated otherwise.

Please review your reference list to ensure that it is complete and correct. If you have cited papers that have been retracted, please include the rationale for doing so in the manuscript text, or remove these references and replace them with relevant current references. Any changes to the reference list should be mentioned in the rebuttal letter that accompanies your revised manuscript. If you need to cite a retracted article, indicate the article’s retracted status in the References list and also include a citation and full reference for the retraction notice.

Attachment Submitted filename: Response to Reviewers.doc

# Editor report: Decision Letter 4

**DOI:** 10.1371/journal.pone.0350983.r009

**Authors:** Swati Jaiswal

22 May 2026

Role and Mechanism of Nitric Oxide-regulated cGAS/STING Pathway-mediated Inflammatory Response in Hypoglycemia-induced Coronary Artery Endothelial Cell Injury

PONE-D-25-31152R4

Dear Dr. Luo,

We’re pleased to inform you that your manuscript has been judged scientifically suitable for publication and will be formally accepted for publication once it meets all outstanding technical requirements.

Within one week, you’ll receive an e-mail detailing the required amendments. When these have been addressed, you’ll receive a formal acceptance letter and your manuscript will be scheduled for publication.

An invoice will be generated when your article is formally accepted. Please note, if your institution has a publishing partnership with PLOS and your article meets the relevant criteria, all or part of your publication costs will be covered. Please make sure your user information is up-to-date by logging into Editorial Manager at Editorial Manager® and clicking the ‘Update My Information' link at the top of the page. For questions related to billing, please contact billing support.

If your institution or institutions have a press office, please notify them about your upcoming paper to help maximize its impact. If they’ll be preparing press materials, please inform our press team as soon as possible -- no later than 48 hours after receiving the formal acceptance. Your manuscript will remain under strict press embargo until 2 pm Eastern Time on the date of publication. For more information, please contact onepress@plos.org.

Kind regards,

Swati Jaiswal

Academic Editor

PLOS One

Additional Editor Comments (optional):

Reviewers' comments:

# Editor report: Acceptance letter

**DOI:** 10.1371/journal.pone.0350983.r010

**Authors:** Swati Jaiswal

PONE-D-25-31152R4

PLOS One

Dear Dr. Luo,

I'm pleased to inform you that your manuscript has been deemed suitable for publication in PLOS One. Congratulations! Your manuscript is now being handed over to our production team.

At this stage, our production department will prepare your paper for publication. This includes ensuring the following:

* All references, tables, and figures are properly cited

* All relevant supporting information is included in the manuscript submission,

* There are no issues that prevent the paper from being properly typeset

You will receive further instructions from the production team, including instructions on how to review your proof when it is ready. Please keep in mind that we are working through a large volume of accepted articles, so please give us a few days to review your paper and let you know the next and final steps.

Lastly, if your institution or institutions have a press office, please let them know about your upcoming paper now to help maximize its impact. If they'll be preparing press materials, please inform our press team within the next 48 hours. Your manuscript will remain under strict press embargo until 2 pm Eastern Time on the date of publication. For more information, please contact onepress@plos.org.

You will receive an invoice from PLOS for your publication fee after your manuscript has reached the completed accept phase. If you receive an email requesting payment before acceptance or for any other service, this may be a phishing scheme. Learn how to identify phishing emails and protect your accounts at https://explore.plos.org/phishing.

If we can help with anything else, please email us at customercare@plos.org.

Thank you for submitting your work to PLOS ONE and supporting open access.

Kind regards,

PLOS ONE Editorial Office Staff

on behalf of

Dr. Swati Jaiswal

Academic Editor

PLOS One
